# Whole-genome sequencing shows modulation of neurodegenerative genes by *Withania somnifera* in human SK-N-SH cells

**DOI:** 10.3389/fnmol.2025.1512727

**Published:** 2025-06-25

**Authors:** Eshita Sharma, Dilip Mehta, Nikita Jadhav, Gunjan Gujrati, S. Dhananya, Manju Moorthy, Gopalakrishna Ramaswamy, Yundong Zhou, Sujit Nair

**Affiliations:** ^1^Phytoveda Pvt. Ltd., Mumbai, India; ^2^Viridis Biopharma Pvt. Ltd., Mumbai, India; ^3^Department of Biological Sciences, The University of Toledo, Toledo, OH, United States; ^4^TheraCUES Innovations Pvt. Ltd., Bengaluru, India; ^5^The Second Department of Oncology, Huizhou First Hospital, Huizhou, Guangdong, China

**Keywords:** *Withania somnifera*, ashwagandha, whole genome sequencing, neurodegenerative disease, aging, healthy aging

## Abstract

**Background:**

Aging is driven by several primary and secondary hallmarks that manifest with age, of which neurodegenerative diseases are important manifestations. The ability to decelerate or reverse aging, and promote healthy aging, has garnered great interest in recent times. In traditional medicine, *Withania somnifera* (WS) or Ashwagandha has been recognized for its adaptogenic and rejuvenative effects.

**Methods:**

To investigate WS-modulated global gene expression profiles, we performed whole-genome sequencing of WS-treated human neuroblastoma SK-N-SH cells at different doses (50 and 100 μg/mL) and time points (3 h and 9 h) and validation by quantitative real-time PCR (qRT-PCR) and immunoblotting. Disease enrichment analysis for brain-related disorders was performed by DisGeNET.

**Results:**

Using differential gene expression analyses, we identified 19,945 WS-modulated genes. Of these, 2,403 and 177 genes were significantly (*p* ≤ 0.05) upregulated and downregulated, respectively, by WS treatment. Interestingly, different patterns of gene expression were exhibited in dose-dependent (9 upregulated, 1 downregulated, 100 μg/mL 3 h vs. 50 μg/mL 3 h; 21 upregulated, 86 downregulated, 100 μg/mL 9 h vs. 50 μg/mL 9 h) and temporal kinetics (210 upregulated, 6 downregulated, 50 μg/mL 9 h vs. 50 μg/mL 3 h; 8 upregulated, 49 downregulated, 100 μg/mL 9 h vs. 100 μg/mL 3 h). Furthermore, qRT-PCR experiments validated the RNA-seq results. WS-modulated genes were implicated in Alzheimer’s disease, migraine, Parkinson’s disease, bipolar disorder, cognition, stress, anxiety, forgetfulness, sleep disorders, and substance abuse among others.

**Conclusion:**

Taken together, our transcriptomic profiling study revealed for the first time that WS may modulate key genes in neurodegenerative disorders with potential beneficial implications for brain-related disorders and healthy aging.

## 1 Introduction

Neurodegenerative disorders (NDs) have been on the rise over the past three decades, affecting approximately 15% of the global population and leading to physical and cognitive disabilities. The growing proportion of elderly individuals worldwide has contributed to an increase in age-related neurological disorders, including dementia, stroke, Alzheimer’s disease, and Parkinson’s disease (Wilson et al., 2023). Currently, more than 3 billion people globally are living with a neurological condition (Steinmetz et al., 2024). People affected with Alzheimer’s disease alone are 40 million worldwide and estimated to rise to 135 million by 2050 (Better, 2023). To overcome these age-linked complications, research has been carried out to understand the underlying mechanisms involved in brain-associated disorders. Chinonin, a flavonoid from *Rhizoma anemarrhena*, has been observed to have neuroprotective activity against brain injury (Hui et al., 2024). In an Alzheimer’s disease rat model, apelin-13 was found to inhibit neuroinflammation by activation of BDNF–TrkB signal transduction (Luo et al., 2019). A co-assembly of cyclodextrin and guanidinium-modified calixarene with insulin decreased synaptic damage and neuronal apoptosis thus improving cognitive function in 5xFAD mice (Zhang et al., 2024).

Recently, medicinal herbal extracts have received greater attention for their major health-beneficial properties and minimal side effects. The Buyang Huanwu decoction has been reported to have beneficial effects on brain hemorrhage by anti-apoptotic mechanisms (Cheng et al., 2024). Using metabolomics analysis, the mechanistic basis for the beneficial effects of the Bushen Tiansui formula in Alzheimer’s disease was found to be due to effects in the cerebral cortex and hippocampus (Li et al., 2022). WS plays an important role in improving mental and physical health to combat diseases and age-related complications and delays the biological aging process. The health attributes of WS include anti-inflammatory, antioxidant, neuroprotection, and antidiabetic activities and increase in muscle strength (Basudkar et al., 2024; Saha et al., 2024). WS exhibits antioxidant property which helps in the prevention of pathogenesis, induced by glycation, thereby reducing various oxidative stress marker levels. WS decreases the pro-inflammatory cytokines, i.e., TNF-*α* and IL-6 which participate in neuroinflammation (Khan et al., 2019). Inflammatory markers *viz.* NF-κB and NLRP3 play a critical function in the progression of neurodegenerative disorders and WS reduces amyloid-beta (Aβ) in IL1β-mediated neuroinflammation in Alzheimer’s disease. The neuroprotective activity of WS root extracts is due to the presence of glycowithanolides which inhibit lipid peroxidation. In addition, withanolides and sitoindosides have been shown to accelerate activities of catalase and glutathione peroxidase (Bashir et al., 2023). Our group performed the preclinical oral GLP toxicity study of WS root extract on Sprague Dawley rats and reported that no-observed-adverse-effect level (NOAEL) of WS root extract was 1,000 mg/kg body weight and safe to use (Wangikar et al., 2024).

Genomics is a powerful tool for understanding the totality of the genetic and environmental factors and their interactions contributing to health and disease (Li et al., 2023). Genome sequencing has become an important component of drug therapeutic interventions. Whole-genome sequencing enables the identification of genes involved in signal transduction pathway in a specific disease condition. Nowadays, genomics is being increasingly used to discover biological pathways along with networks triggering complex diseases (Kabir et al., 2018). We have previously reported the pharmacogenomics of personalized medicine for cancer ethnicity (Nair and LLerena, 2018), sulforaphane (Khor et al., 2006; Nair and Kong, 2016), prostate cancer (Nair Sujit, 2014; Nair and Tony Kong, 2015), antioxidant butylated hydroxyanisole (Nair et al., 2006), soy isoflavone (Barve et al., 2008), and toxicogenomics of tunicamycin (Nair et al., 2007).

A number of research projects highlighted the clinical potential of high-throughput sequencing technologies in various types of cancers (Fransson et al., 2020; Przybyła et al., 2022). Various studies have demonstrated the potential neuroprotective role of WS in neuroblastoma cell lines (Kumar et al., 2010; Kurapati et al., 2013; Atluri et al., 2020; Siddiqui et al., 2021; Wongtrakul et al., 2021). However, these reports are based on enzymatic markers using WS plant parts or bioactives present in WS. RNA-sequencing studies including the effect of WS extracts on dysregulated gene expression and elucidation of key biomarkers involved in neurodegenerative disorders are still lacking. Hence, the present study was designed to investigate the neuroprotective effect of WS extract using a WS-treated SK-N-SH neuroblastoma cell line *via* whole-genome sequencing at different time points and dose concentrations. RNA-sequencing and comprehensive bioinformatics tools were used to illustrate the role of WS in various molecular functions and biological processes involved in brain-related disorders. Furthermore, key biomarkers involved in anti-neuroinflammation and antioxidant signal transduction at a transcriptional level were also elucidated along with functional biological networks implicated in WS modulation of transcriptomic markers.

## 2 Materials and methods

### 2.1 *Withania somnifera* extract

A standardized root extract containing 2.5% *Withania somnifera* (LongeFera™) was provided by Phytoveda Pvt. Ltd., Mumbai, India. The samples were previously standardized for withanolide and withanoside content using HPLC-PDA as reported earlier by Wangikar et al. (2024).

### 2.2 Cell culture and treatment

Human neuroblastoma SK-N-SH cells were obtained from American Type Cell Culture (ATCC), USA. The cells were retained in Minimum Essential Medium Eagle (EMEM) which was supplemented with fetal bovine serum (15% v/v), penicillin (100 U/mL), and streptomycin (100 μg/mL). In six-well plates (Corning, USA), the cells were plated at a density of 1.5 × 10^4^ cells/well and incubated in a CO_2_ incubator with 5% relative humidity at 37°C. Furthermore, the cells were starved overnight in EMEM containing antibiotics and 0.5% fetal bovine serum. Cells were further treated with WS extract at two concentrations *viz.* 50 μg/mL and 100 μg/mL at two time points, i.e., 3 and 9 h to capture temporal kinetics of RNA expression. For Western blotting, cells were treated with liposaccharide (LPS, 1 μg/mL) for 3 h followed by treatment with different concentrations of WS.

### 2.3 RNA extraction, cDNA library preparation, and sequencing

The isolation of total RNA was performed using Total Aurum RNA Mini Kit (Bio-Rad, USA) as per the instructions of the manufacturer followed by quality and quantity measurement using NanoDrop (DeNovix, Thermo Scientific, USA) and Agilent Screen TapeStation system (Agilent Technologies, USA). Qubit High Sensitivity assay showed good concentration of RNA in all samples. The Agilent TapeStation profiles showed that RNA had good integrity with RIN values between 9.3 and 9.8 for all samples. For cDNA library preparation, RNA of each sample was taken using the NEB Ultra II directional RNA-Seq Library Prep kit (New England Biolabs, USA). The quantification of prepared libraries was completed using Qubit HS assay (Invitrogen, USA). The diluted libraries were loaded onto the flow cell for paired end (PE) sequencing using Illumina NovaSeq (Illumina, San Diego, CA).

### 2.4 Differential expression analysis and functional enrichment analysis

For the detection of differentially expressed transcripts among treated and untreated cell lines, sample-specific filtered reads were mapped to reference the House genome using STAR (v.2.2.11a). The raw read counts were determined using featureCounts (2.0.1), and count data was normalized employing DESeq2. Differential expressions were performed in 50 μg/mL 3 h vs. control 3 h (50_3h vs. C_3h), 100 μg/mL 3 h vs. control 3 h (100_3h vs. C_3h), 50 μg/mL 9 h vs. control 9 h (50_9h vs. C_9h), 100 μg/mL 9 h vs. control 9 h (100_9h vs. C_9h), 100 μg/mL 3 h vs. 50 μg/mL 3 h (100_3h vs. 50_3h), and 100 μg/mL 9 h vs. 50 μg/mL 9 h (100_9h vs. 50_9h). Transcripts with log2 FC ≥ 2, log2 FC ≤ −2, and *p* ≤ 0.05 were considered as upregulated or downregulated, respectively. The Benjamini–Hochberg false discovery rate (FDR < 0.05) was set at 5%. Furthermore, functional enrichment analysis comprising biological process, molecular function, gene ontology, cellular components, and Reactome pathway enrichment analysis (over-representation method) was performed for the differentially expressed significant genes using clusterProfiler R package.

### 2.5 Disease enrichment analysis

Disease enrichment analysis was performed using DisGeNET. Disease enrichment analyses including disease gene analysis and disease ontology were performed for differentially expressed significant genes. Transcripts with positive or negative log *p*-value (*p* ≤ 0.05) were considered upregulated or downregulated, respectively. The Benjamini–Hochberg false discovery rate (FDR < 0.05) was set at 5%.

### 2.6 Quantitative real-time polymerase chain reaction (qRT-PCR) analyses

To validate the RNA-seq data, genes were selected to check the relative expression levels measured using qRT-PCR. The first strand cDNA was synthesized from 4 μg of DNaseI-treated total RNA using a high-capacity cDNA QuantiTect Reverse Transcription kit (Qiagen, USA). To design the gene-specific primers for qRT-PCR, Primer Express 3.0.1 software (Applied Biosystems, USA) was used (Table 1). *β*-actin was used as a housekeeping gene. Furthermore, qRT amplification was carried out on a 384-well QuantStudio real-time PCR machine (Applied Biosystems, USA) using the SYBR Green qPCR Master Mix (Thermo Scientific, USA) intercalating dye chemistry. The conditions used for qRT-PCR were 95°C for 10 min followed by 50 cycles of 95°C for 15 s, 55°C for 30 s, and 72°C for 30 s (data collection). Melting curve analysis was performed to check for the absence of primer dimers. The relative expression of each gene was estimated using the 2^-ΔΔCt^ method (Kumar et al., 2016).

**Table 1:** List of primer sequences of selected transcripts used for quantitative reverse transcriptase-polymerase chain reaction (qRT-PCR) analysis.

S. no	Gene name	Gene symbol	NCBI accession		Primer sequence
1	Glutathione specific gamma glutamylcyclotransferase 1	*CHAC1*	NM_024111.6	Forward	5'-GTGCTTGGTGGCTACGATACC-3'
				Reverse	5'-TCAGTGGTTGGTCAGGAGCAT-3'
2	Heme oxygenase 1 gene	*HMOX1*	NM_002133.3	Forward	5'-GGGTGATAGAAGAGGCCAAGACT-3'
				Reverse	5'-AGCTCCTGCAACTCCTCAAAGA-3'
3	Gamma-glutamyltransferase light chain 1	*GGTLC1*	NM_178311.3	Forward	5'-AACCTCTACTTTGGCTCCAAGGT-3'
				Reverse	5'-GCTGAAGTCATCCATTTCATTATTGA-3'
4	NLR family pyrin domain-containing 2B	*NLRP2B*	NM_001319967.1	Forward	5'-ACTTCAACCTGCAGGCTCTTCT-3'
				Reverse	5'-GATCAGAGACTTGAACTTGCACAAG-3'
5	Solute carrier family 7-member 11	*SLC7A11*	NM_014331.4	Forward	5'-CCTCTTCATGGTTGCCCTTTC-3'
				Reverse	5'-ATGACGAAGCCAATCCCTGTA-3'
6	Synuclein alpha interacting protein	*SNCAIP*	DQ227317.1	Forward	5'-AAGACAAAGATAAGGGCAGGACTCT-3'
				Reverse	5'-TTGATCCCCCGATTCGTTAC-3'
7	Nitric oxide synthase	*NOS1*	U17327.1	Forward	5'-GCTCCTTAGCCGTCAAAACCT-3'
				Reverse	5'-GTGGAGACGCACGAAGATAGTTG-3'
8	Neuropeptide Y receptor Y4-2	*NPY4R2*	NM_001278795.2	Forward	5'-GGTAACCTCTGCCTGATGTGTGT-3'
				Reverse	5'-GGTTGGTCACGTTGGCTTTC-3'
9	Transmembrane protein 100	*TMEM100*	NM_001099640.2	Forward	5'-ATGGCAGCGACGATGGA-3'
				Reverse	5'-CAGAGGGACTGTGGTGATCACA-3'
10	β-actin	*ACTB*	PQ040393.1	Forward	5'-CCAACTGGGACGACATGGA-3'
				Reverse	5'-AGCCACACGCAGCTCATTG-3'

### 2.7 Protein extraction and western blotting

For protein extraction, cells were harvested and lysed in a Protease Inhibitor Cocktail (Abcam, USA) with RIPA buffer and centrifuged at 16,000 × *g* for 20 min at 4°C. The supernatant was collected, and protein concentration was measured by Pierce™ BCA Protein Assay Kits (Thermo Fisher Scientific, USA) using bovine serum albumin as standard.

For Western blotting, protein samples (20 μg protein/lane per sample) were dissolved in Laemmli sample buffer 2 × and separated on Mini-PROTEAN® Precast Gels (4–20%) (Bio-Rad, USA). Following this, proteins were transferred to Immun-Blot® PVDF Membrane (Bio-Rad, USA) after SDS-polyacrylamide gel electrophoresis and membranes were incubated in a 5% bovine serum albumin blocking buffer for about 2 h. After blocking, the membranes were incubated with primary antibodies overnight at 4°C: including anti-*β*-actin, anti-total NF-kB, anti-heme oxygenase-1 enzyme (HO-1) (1:1000); anti-TNF-*α* (1:500); and anti-phospho-NF-kB (1:250). All antibodies were obtained from Cell Signaling Technology, USA. Furthermore, the membranes were incubated with specific secondary antibodies (anti-rabbit IgG, anti-mouse IgG HRP-linked antibodies, 1:5000) for 1 h at room temperature. The protein bands were developed using SuperSignal™ West Femto Maximum Sensitivity Substrate (Thermo Fisher Scientific, USA), visualized with ChemiDoc MP imaging system (Bio-Rad, USA), and analyzed using ImageJ software. The protein band intensity was normalized to β-actin and expressed as the ratio of control.

### 2.8 Statistical analyses

All data were given as mean ± standard deviation (SD). Statistical analyses were performed using SigmaPlot (version 15.0.0.13). The significance in treatment effects was analyzed using one-way analysis of variance (ANOVA) with Duncan’s multiple range tests for *post-hoc* comparisons using SigmaPlot. Comparisons between the two treatments were performed using an unpaired *t*-test. A *p* < 0.05 was considered statistically significant.

## 3 Results

### 3.1 Reads generation and homology search

High-throughput sequencing of *Withania*-treated SK-N-SH human neuroblastoma cells (17 samples) resulted in 855,778,419 paired end reads. Total reads were reduced to 849,906,890 paired end reads representing 123,194,268 (74.48%) for 50 μg/mL_3h samples; 133,473,085 (77.46%) for 100 μg/mL_3h samples; 141,443,937 (68.35%) for 50 μg/mL_9h samples; and 163,215,314 (59.95%) for 100 μg/mL_9h samples (Table 2).

**Table 2:** Details of various samples taken in the study and read sequence information.

Sample description	Total paired reads before quality filtering	Total paired reads after quality filtering	% Quality reads
Control (1) 3 h	43,137,615	42,864,906	99.37
Control (2) 3 h	50,585,646	50,347,089	99.53
Control (3) 3 h	46,568,472	46,313,874	99.45
50 μg/mL (1) 3 h	37,441,555	37,205,410	99.37
50 μg/mL (2) 3 h	45,003,794	44,619,233	99.15
50 μg/mL (3) 3 h	41,621,925	41,369,625	99.39
100 μg/mL (1) 3 h	42,331,808	42,014,817	99.25
100 μg/mL (2) 3 h	45,215,816	44,911,764	99.33
100 μg/mL (3) 3 h	46,967,427	46,546,504	99.10
Control (1) 9 h	51,887,358	51,490,023	99.23
Control (2) 9 h	50,135,019	49,840,427	99.41
Control (3) 9 h	48,126,121	47,723,967	99.16
50 μg/mL (1) 9 h	56,410,234	56,003,138	99.28
50 μg/mL (2) 9 h	27,281,887	27,058,261	99.18
50 μg/mL (3) 9 h	58,864,017	58,382,538	99.18
100 μg/mL (1) 9 h	58,177,880	57,730,234	99.23
100 μg/mL (2) 9 h	50,778,685	50,548,101	99.55
100 μg/mL (3) 9 h	55,243,160	54,936,979	99.45

### 3.2 Differential gene expression analyses

**Figure 1:**
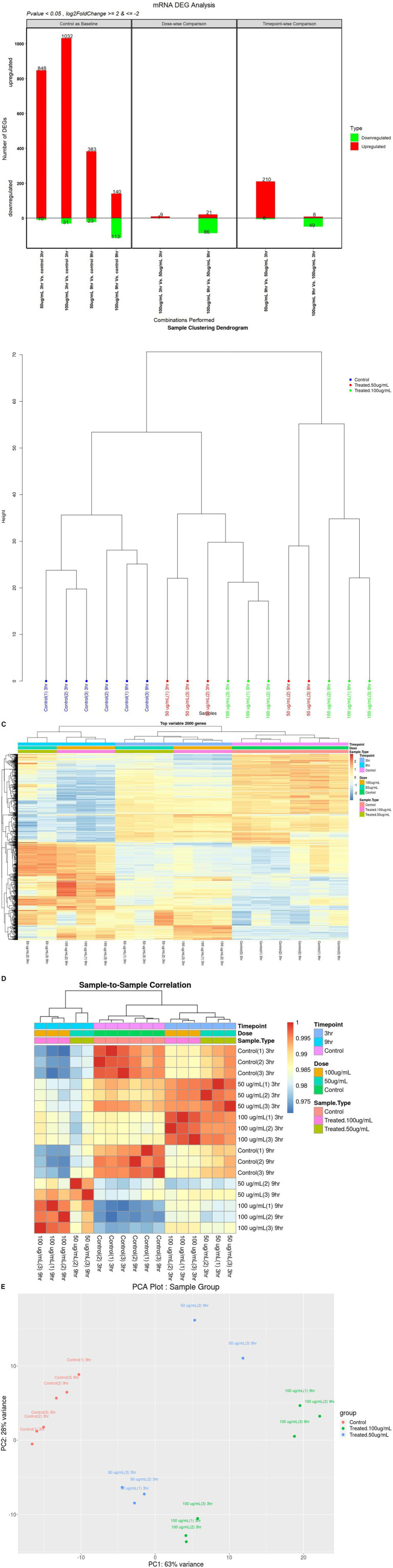
Differential gene expression (DGE) mRNA analyses: **(A)** up- or downregulation of genes in WS-treated human neuroblastoma SK-N-SH cells at various treatments; **(B)** sample clustering dendrogram; **(C)** heatmap of top variable 2000 genes; **(D)** sample-to-sample correlation; **(E)** PCA plot of sample group.

To measure differential gene expression (DGE), high-quality reads were mapped to assembled transcripts from the reference genome (Supplementary Table 1). A total of 19,945 genes were expressed after data normalization. Among these genes, 38.49% were upregulated and 13.41% were downregulated. In differential gene expression analysis, in comparison with the control, we identified 848 upregulated genes and 10 downregulated genes in 50_3h vs. C_3h, 1,032 upregulated genes and 31 downregulated genes in 100_3h vs. C_3h, 383 upregulated genes and 23 downregulated genes in 50_9h vs. C_9h, and 140 upregulated genes and 113 downregulated genes in 100_9h vs. C_9h. Furthermore, comparison of differentially expressed genes (DEGs) at different doses showed upregulation of 9 genes and downregulation of 1 gene in 100_3h vs. 50_3h, whereas 21 genes were upregulated, and 86 genes were downregulated in 100_9h vs. 50_9h. In addition, the comparison of DEGs at different time points (temporal kinetics) showed 210 upregulated genes and 6 downregulated genes (50_9h vs. 50_3h) as well as 8 upregulated genes and 49 downregulated genes in 100_9h vs. 100_3h. Overall, 2,697 genes were either elevated (2403) or suppressed (177) in WS-treated SK-N-SH cells at different time points and doses (Figures 1A–E).

#### 3.2.1 Upregulated differentially expressed genes (DEGs)

Functional enrichment-based analysis revealed that the upregulated genes were classified into categories, including protein coding genes, cardiomyopathy genes, antioxidant genes, heat shock proteins, phosphatases, dehydrogenases, transcription factors, apoptosis, cell proliferation, differentiation and mitogenesis, and G-protein coupled receptor 1.

Gene expression in SK-N-SH cells by WS treatment resulted in the upregulation of genes involved in the prevention of various brain-related disorders. The category of transcriptional factors and interacting partners predominated the upregulated genes followed by dehydrogenases and phosphatases. Among these, histone proteins (H2AX, H2BY), heat shock proteins (HSP family), and apoptotic genes were upregulated. Furthermore, the upregulated antioxidant genes possibly promote ROS scavenging and maintaining protein functional conformation in treated neuroblastoma cells.

Ion transporters play crucial roles in the regulation of ionic homeostasis and cell signaling under physiological conditions (Bao et al., 2022). Transcripts encoding ion transporters (NFERF1) were expressed in treated SK-N-SH cells. The solute carrier family constitutes abundant amino acid transporters which contribute to assisting amino acid transfer across cell membranes. Among the amino acid transporters, higher expression of SLC7A11 reportedly mediates cysteine uptake and simultaneous efflux of glutamate (Jakobsen and Nielsen, 2024).

Aquaporins comprised of water channel proteins family are present in plasma membranes of diverse cells and facilitate the water flow across the cell membranes responsive to osmotic gradient against oxidative stress (Papadopoulos and Saadoun, 2015). Aquaporins (AQP4, AQP9, AQP12A, AQP12B, and isoforms) were upregulated in WS-treated cells supporting their involvement in controlling cellular motility mechanisms in the cells.

Tight junctions are intercellular protein complexes that regulate cell polarity and paracellular permeability to maintain tissue integrity and homeostasis (Nehme et al., 2023). Tight junction proteins, i.e., claudin (CLDN2, CLDN8, CLDN1, CLDN16, CLDN17, and CLDN19), were upregulated in treated SK-N-SH cells.

**Figure 2:**
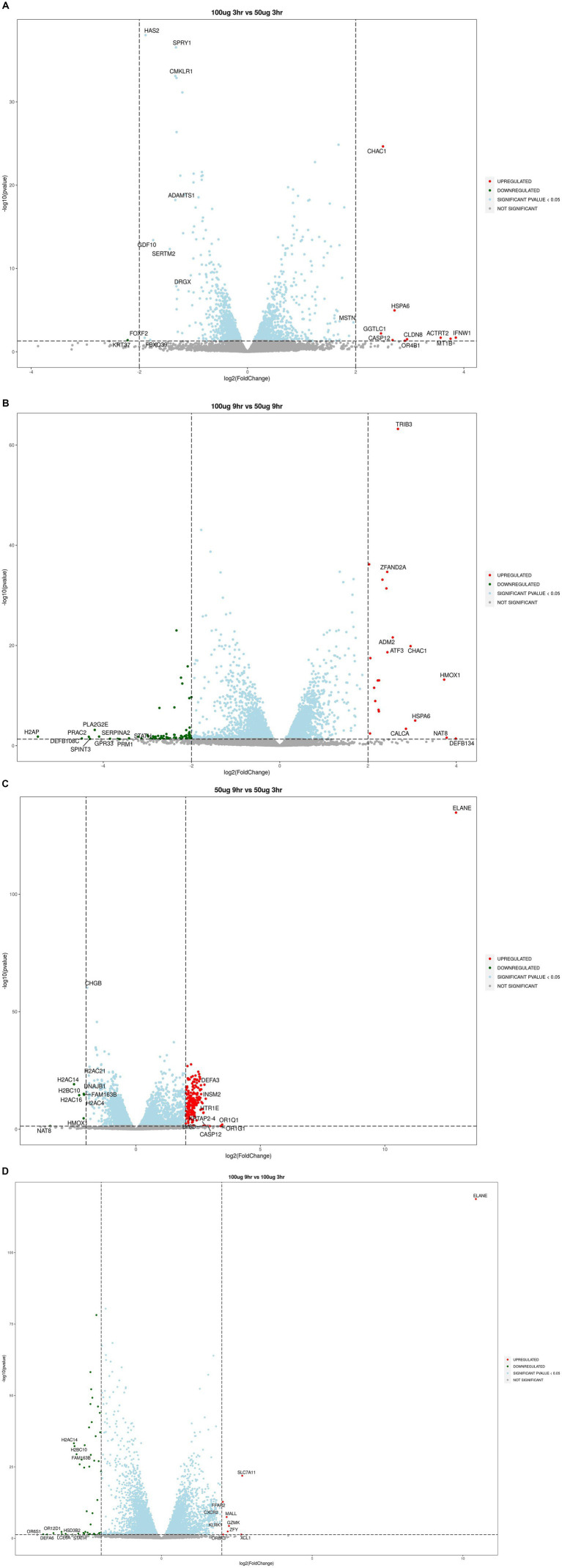
Volcano plots showing upregulated or downregulation of genes at different time points and dose concentrations: **(A)** 100 μg/mL_3h vs. 50 μg/mL_3h; **(B)** 100 μg/mL_9h vs. 50 μg/mL_9h; **(C)** 50 μg/mL_9h vs. 50 μg/mL_3h; **(D)** 100 μg/mL_9h vs. 100 μg/mL_3h.

The upregulated genes included tumor suppressor MT1B (metallothionein 1B), inflammatory caspase CASP12 (caspase 12), glutathione metabolism NLR (gamma-glutamyltransferase light chain 1), CLDN8 (claudin-8), oxidative stress gene HMOX1, CALCA (calcitonin related polypeptide alpha), DDIT3 and DDIT4 (DNA damage-inducible transcripts 3 and 4), proapoptotic compounds ARC (activity regulated cytoskeleton associated protein), ADM2 (adrenomedullin 2), cytoskeleton organization ACTRT2 (actin related protein T2), and serine protease ELANE (elastase, neutrophil expressed) (Figures 2A–D).

#### 3.2.2 Downregulated DEGs

Among the downregulated genes, the neurotoxicity-associated genes included nitric oxide synthase (NOS1), a disintegrin and metalloproteinase with thrombospondin motifs 1 (ADAMTS1), colony-stimulating factor 2 (CSF2), nuclear factor erythroid-derived 2- like 2 (NFE2l2), hypocretin receptor 2 (HCRTR2), dopamine beta-hydroxylase (DBH), 5-hydroxytryptamine receptor 1E (HTR1E), chromogranin A and B (CHGs), achaete-scute homolog 1 (ASCL1), solute carrier family 5 member 7 (SLC5A7), C-X-C motif chemokine ligand 12 (CXCL12), GDNF family receptor alpha-2 (GFRA2), transmembrane protein 100 (TMEM100), neurogenin 2 (NEUROG2), prokineticin receptor 2 (PROKR2), H2A. P histone (H2AP), H2B clustered histone 10 (H2BC10), PRAME family member 12 (PRAMEF12), neuropeptide Y receptor-2R (NPY2R), and insulinoma-associated 2 gene (INSM2) were dominantly expressed (Figures 2A–D).

**Figure 3:**
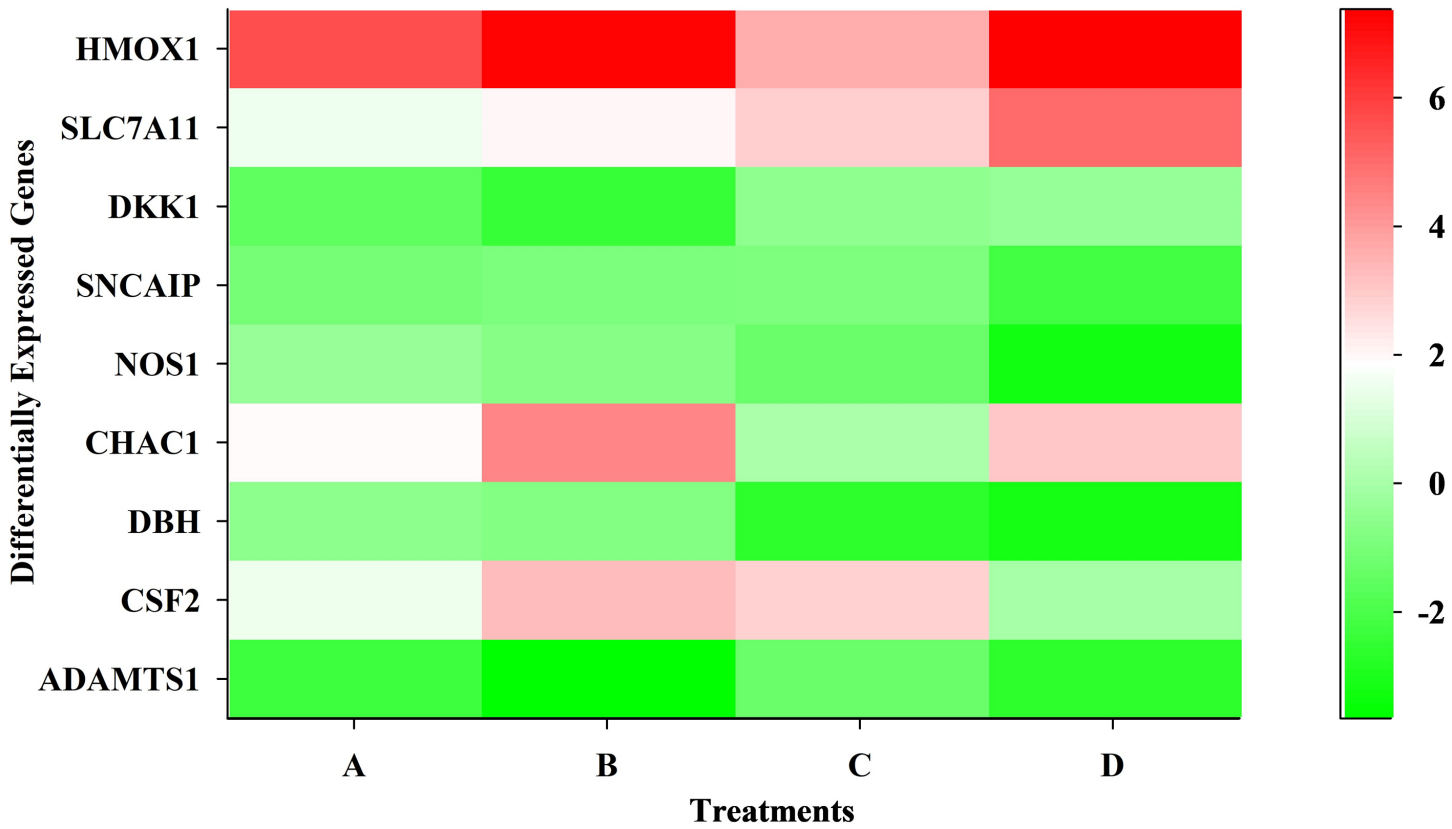
Heatmap showing differentially expressed genes at different time points and dose concentrations.

The ATP-binding cassette transporters (ABCC1, KCNJ family), transcription factors (TFAP2B), G-protein coupled receptors (BDKRB), and chemokine receptor (CXCL12) expressions were downregulated in treated SK-N-SH cells. Figure 3 is a heat map showing differentially expressed genes at different time points and concentrations.

### 3.3 Gene ontology (GO) and Reactome analysis

GO and Reactome enrichment analyses were performed to significantly understand the key pathways enriched in WS-treated SK-N-SH cells (Supplementary Figures S1–S7). Among the enriched cellular processes, secretory granule lumen complex, vesicle lumen complex, aggresome, apical plasma membrane complex, nucleosome complex, DNA packaging complex, protein–DNA complex, and transport vesicle complex were enriched in treated SK-N-SH cells. In biological processes, response to unfolded proteins, response to topologically incorrect proteins, response to heat, response to temperature stimulus, detection of chemical stimuli involved in sensory and smell perception, protein folding, and intrinsic apoptotic pathway triggered in response to endoplasmic reticulum stress were enriched in WS-treated cells. Interestingly, positive regulation of proteasomal ubiquitin-dependent protein catabolic process and chaperone-mediated protein folding cellular response to topologically incorrect protein were enriched in treated SK-N-SH cells. Moreover, protein folding chaperons, misfolded, unfolded, and heat shock protein binding, ATP-dependent protein folding chaperones, amino acid, carboxylic acid, and organic acid transmembrane transport activity, and oxidoreductase activity were enriched in molecular function in the case of WS-treated cells at different concentrations. Interestingly, ubiquitin and ubiquitin-like protein ligase protein binding, receptor ligand activity, and transcription corepressor activity were predominantly enriched molecular function. Furthermore, cellular response to heat and chemical stress, response to EIF2AK4 (GCN2) to amino acid deficiency, cellular response to starvation, KEAP1-NFE2L2 pathway, nuclear events mediated by NFE2L2, regulation of HSF1-mediated heat shock response, NFE2L2 regulating antioxidant/detoxification enzymes, ATF4-activated genes in response to endoplasmic reticulum stress and transport of inorganic cations, amino acids, and oligopeptides were enriched Reactome pathways.

### 3.4 Disease enrichment analysis

**Figure 4:**
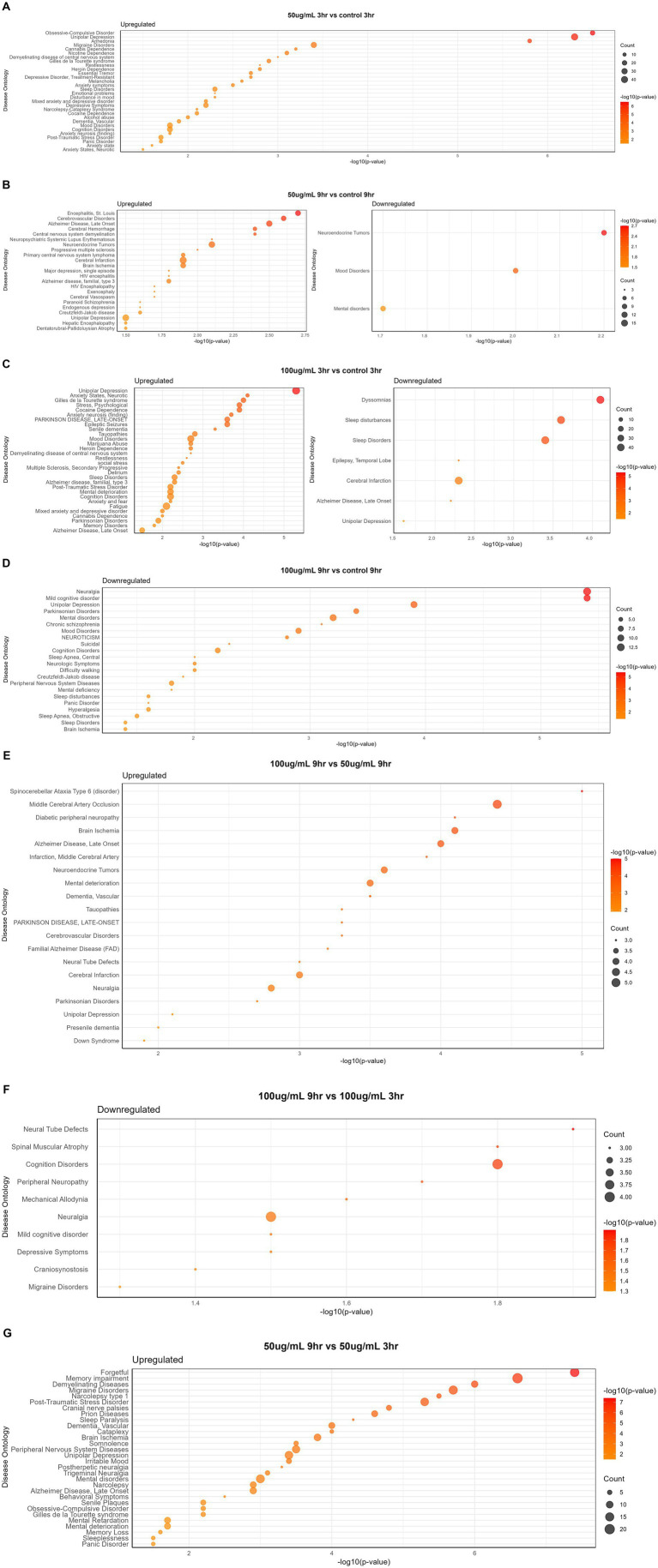
Disease-specific enrichment analysis of WS-treated neuroblastoma cells at different time points and concentration: **(A)** 50 μg/mL_3h vs. control_3h; **(B)** 50 μg/mL_9h vs. control_9h; **(C)** 100 μg/mL_3h vs. control_3h; **(D)** 100 μg/mL_9h vs. control_9h; **(E)** 100 μg/mL_9h vs. 50 μg/mL _9h; **(F)** 100 μg/mL_9h vs. 100 μg/mL _3h; **(G)** 50 μg/mL_9h vs. 50 μg/mL _3h.

**Figure 5:**
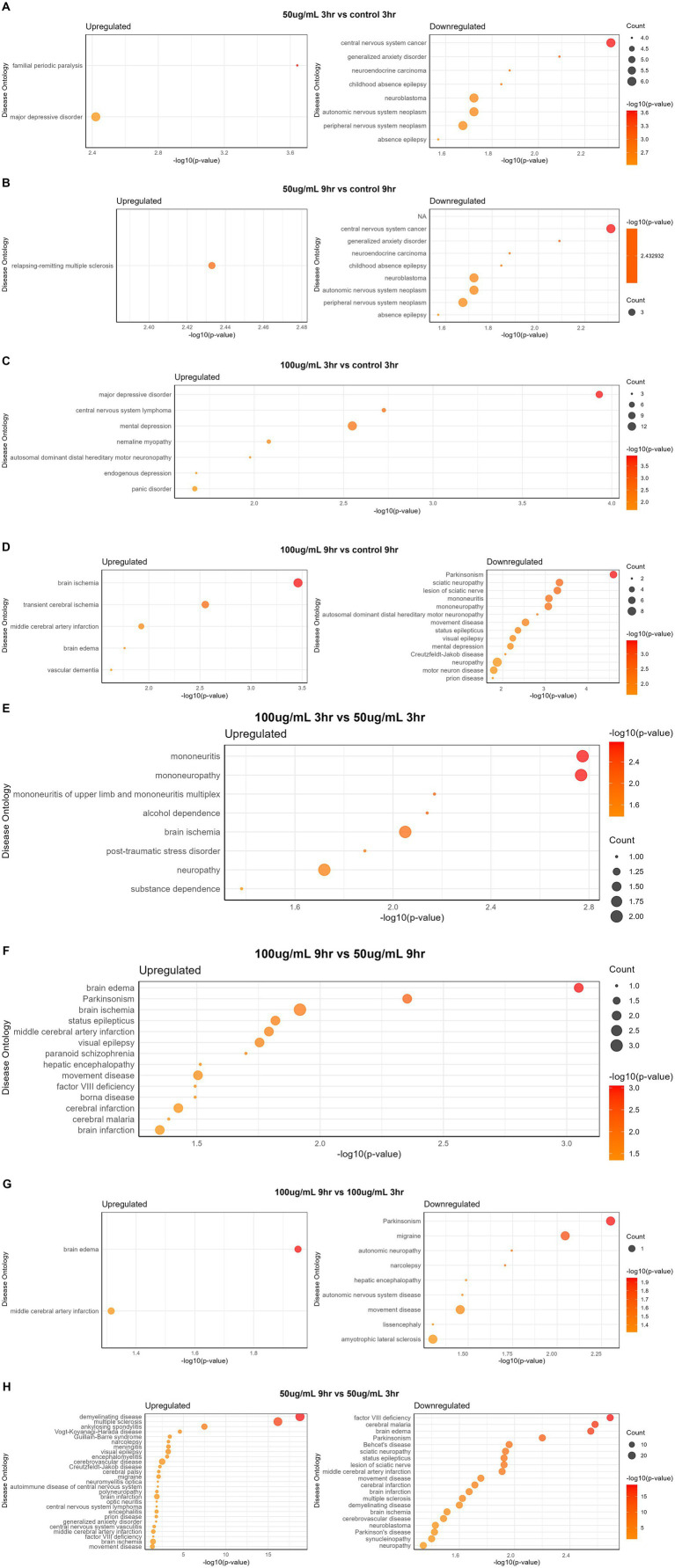
Disease ontology analysis of WS-treated SK-N-SH cells at different time points and concentration: **(A)** 50 μg/mL_3h vs. control_3h; **(B)** 50 μg/mL_9h vs. control_9h; **(C)** 100 μg/mL_3h vs. control_3h; **(D)** 100 μg/mL_9h vs. control_9h; **(E)** 100 μg/mL_3h vs. 50 μg/mL _3h; **(F)** 100 μg/mL_9h vs. 50 μg/mL _9h; **(G)** 100 μg/mL_9h vs. 100 μg/mL _3h; **(H)** 50 μg/mL_9h vs. 50 μg/mL _3h.

The disease gene (DisGeNET) and gene ontology (GO) analyses were carried out to evaluate the WS effect on disease gene interactions, specifically related to brain disorders (Supplementary Tables S2–S4; Figures 4A–G, 5A–H). Genes related to various brain disorders were found to be upregulated or downregulated on various treatments at different time points (Tables 3, 4).

**Table 3:** Downregulated genes at different time points and dose concentrations after WS treatment in brain-related disorders.

Gene name	Gene symbol	Accession number	Disease	Fold change			
				50_3h vs. C_3h	50_9h vs. C_9h	100_3h vs. C_3h	100_9h vs. C_9h
Insulinoma-associated 2 gene	INSM2	NM_032594.4	Central nervous system cancer	1.85	−2.52	−4.86	−4.42
Achaete-scute homolog 1	ASCL1	NM_004316.4	−2.4	−2.78	−3.69	−3.97
Prokineticin receptor 2	PROKR2	NM_144773.4	Generalized anxiety disorder	−3.12	−2.56	−3.56	−4.41
5-hydroxytryptamine receptor 1E	HTR1E	NM_000865.3	−2.14		−2.19	−2.2
Achaete-scute homolog 1	ASCL1	NM_004316.4	Neuroblastoma, autonomic and peripheral nervous system neoplasm,	−2.4	−2.78	−3.69	−3.97
Neuropeptide Y receptor Y2	NPY2R	NM_000910.4	−2.54	−1.26	−2.74	−2.88
Neuropeptide Y receptor Y2	NPY2R	NM_000910.4	Childhood absence epilepsy	−2.54	−1.26	−2.74	−2.88
ADAM metallopeptidase with thrombospondin type 1 motif 1	ADAMTS1	NM_006988.5	−2.26	−1.31	−3.59	−2.64
Dopamine beta-hydroxylase	DBH	NM_000787.4	−0.56	−2.64	−0.78	−3.15
Vascular endothelial growth factor-D	VEGFD	NM_004469.5	−0.32	−2.2	−0.39	−1.72
Chromogranin A	CHGA	NM_001275.4	−0.56	−1.93	−0.93	−2.96
Chromogranin B	CHGB	NM_001819.3	−0.68	−3.32	−0.76	−3.08
ATP-binding cassette subfamily C member 8	ABCC8	NM_001287174.3	Parkinsonism, sciatic neuropathy, lesion of sciatic nerve, mononeuropathy	−0.62	−1.38	−0.98	−2.07
Potassium inwardly rectifying channel, subfamily J, member 8	KCNJ8	NM_004982.4	−1.59	−1.61	−2.41	−3.08
Potassium inwardly rectifying channel, subfamily J, member 11	KCNJ11	NM_000525.4	−1.31	−1.48	−1.32	−2.06
GDNF family receptor alpha 2	GFRA2	NM_001495.5	−0.51	−1.74	−0.77	−2.62
Bradykinin receptor B1	BDKRB1	KJ950627.1	−1.56	−0.83	−2.04	−2.17
Bradykinin receptor B2	BDKRB2	AY275465.1	−0.79	−1.1	−1.14	−2.46
Solute carrier family 5 member 7	SLC5A7	BC111524.1	Mental depression	−0.53	−1.02	−0.65	−2.02
Solute carrier family 5 member 7	SLC5A7	BC111524.1	−0.53	−1.02	−0.65	−2.02
Neuropeptide Y receptor Y2	NPY2R	NM_000910.4	−2.54	−1.26	−2.74	−2.88
Dopamine beta hydroxylase	DBH	NM_000787.4	−0.56	−2.64	−0.78	−3.15
C-X-C motif chemokine ligand 12	CXCL12	NM_199168.4	−0.89	−1.1	−1.02	−2.16
ATP-binding cassette subfamily C member 8	ABCC8	NM_001287174.3	Visual epilepsy	−0.62	−1.38	−0.98	−2.07
ADAM Metallopeptidase With Thrombospondin Type 1 Motif 1	ADAMTS1	NM_006988.5	−2.26	−1.31	−3.59	−2.64
Potassium inwardly rectifying channel, subfamily J, member 11	KCNJ11	NM_000525.4	−1.31	−1.48	−1.32	−2.06
ATP-binding cassette subfamily C member 8	ABCC8	NM_001287174.3	Movement disease	−0.62	−1.38	−0.98	−2.07
Potassium inwardly rectifying channel, subfamily J, member 8	KCNJ8	NM_004982.4	−1.59	−1.61	−2.41	−3.08
Potassium inwardly rectifying channel, subfamily J, member 11	KCNJ11	NM_000525.4	−1.31	−1.48	−1.32	−2.06
GDNF family receptor alpha 2	GFRA2	NM_001495.5	−0.51	−1.74	−0.77	−2.62
Chromogranin A	CHGA	NM_001275.4	Motor neuron disease	−0.56	−1.93	−0.93	−2.96
Chromogranin B	CHGB	NM_001819.3	−0.68	−3.32	−0.76	−3.08
Solute carrier family 5 member 7	SLC5A7	BC111524.1	−0.53	−1.02	−0.65	−2.02
Chromogranin A	CHGA	NM_001275.4	Prion disease	−0.56	−1.93	−0.93	−2.96
Chromogranin B	CHGB	NM_001819.3	−0.68	−3.32	−0.76	−3.08

**Table 4:** Upregulated genes at different time points and dose concentrations after WS treatment in brain-related disorders.

Gene name	Gene symbol	Accession number	Disease	Fold change			
				50_3h vs. C_3h	50_9h vs. C_9h	100_3h vs. C_3h	100_9h vs. C_9h
Heme oxygenase 1	HMOX1	NM_002133.3	Cerebral infarction	5.644	3.594	7.271	7.321
Activity-regulated cytoskeleton-associated protein	ARC	AF193421.1	4.347	1.855	2.538	1.238
Heme oxygenase 1	HMOX1	NM_002133.3	Brain edema	5.644	3.594	7.271	7.321
Solute carrier family 7 member 11	SLC7A11	NM_014331.4	1.518	2.906	2.029	5.044
Heme oxygenase 1	HMOX1	NM_002133.3	Parkinsonism, movement disease	5.644	3.594	7.271	7.321
Heat shock protein family A	HSPA1A	NM_005345.6	4.756	2.851	6.42	5.097
Calcitonin related polypeptide alpha	CALCA	NM_001033952.3	Brain ischemia, transient cerebral ischemia	1.683	-	3.133	2.476
Protein phosphatase 1 regulatory subunit 15A	PPP1R15A	NM_014330.5	0.654	0.43	1.848	2.15
Heat shock protein family A member 8	HSPA8	NM_153201.4	1.345	1.487	1.765	2.382
Quinone-oxidoreductase-1	NQO1	NM_000903.3	0.984	1.862	1.092	2.459
Elastase neutrophil expressed	ELANE	NM_001972.4	1.387	13.13	1.998	11.313
DNA damage-inducible transcript 3	DDIT3	NM_001195053.1	1.416	0.553	3.099	2.881
Metallothionein 1H	MT1H	NM_005951.2	1.174	5.255	–	4.008
Heat shock protein family B	HSPA1B	NM_005346.6	4.784	2.678	6.391	4.917
Metallothionein 1B	MT1B	NM_005947.3	Mononeuropathy, Mononeuritis	–	1.68	–	–
Caspase 12	CASP12	NM_001191016.3	0.163	2.753	2.845	–
Opioid Receptor Kappa 1	OPRK1	AF498922.1	Drug dependence, heroin dependence, opiate dependence,	2.078	1.383	2.093	–
Gamma-glutamyltransferase light chain 1	GGTLC1	NM_178311.3	Alcohol dependence	0.411	1.586	2.879	–
Gamma-aminobutyric acid receptor subunit α-2	GABRA2	NM_000807.4	Substance dependence	2.203	1.016	2.097	–
Potassium inwardly rectifying channel subfamily J member 6	KCNJ6	NM_002240.5	2.115	1.165	1.947	0.412
Proenkephalin	PENK	NM_001135690.3	2.087	0.642	–	–
Leptin	LEP	NM_000230.3	2.156	0.585	2.776	0.971
Phospholipase A2 group IIA	PLA2G2A	NM_000300.4	2.883	2.199		0.941
Gamma-aminobutyric acid type B receptor subunit 2	GABBR2	NM_005458.8	2.066	1.161	1.927	0.352
Apolipoprotein C-III	APOC3	NM_000040.3	4.195	0.57	4.419	-
Sodium voltage-gated channel α subunit 4	SCN4A	NM_000334.4	2.014	1.381	2.208	0.734
Melanocortin-2 receptor	MC2R	NM_000529.2	2.321	0.3	2.569	0.141
Apolipoprotein A-IV	APOA4	NM_000482.4	Major depressive disorder	4.195	2.689	3.504	0.988
Interleukin 5	IL5	NM_000879.3	2.325	1.132	3.277	1.79
Interleukin 10	IL10	NM_000572.3	1.99	1.265	2.035	0.443
Interleukin 3	IL3	NM_000588.4	1.979	1.241	3.121	1.561
Tryptophan hydroxylase isoform 2	TPH2	NM_173353.4	2.037	1.845	1.877	1.172
Cholinergic receptor nicotinic β 3 subunit	CHRNB3	NM_004198.3	3.294	-	2.606	-
Corticotrophin-releasing hormone	CRH	NM_000756.4	1.739	1.43	3.006	1.288
Cysteinyl leukotriene receptor 1	CYSLTR1	NM_001282187.2	2.195	1.094	3.877	0.341
5-hydroxytryptamine	HTR1A	NM_000524.4	2.661	1.904	2.42	1.288
Heat shock protein family A member 1 A	HSPA1A	NM_005345.6	4.756	2.851	6.42	5.097
Heat shock protein family A member 1 like	HSPA1L	NM_005527.4	1.513	0.919	2.044	1.949
Tachykinin precursor 1	TAC1	NM_013996.3	Obstructive sleep apnea, sleep disorder	2.168	0.825	2.429	0.403
Interleukin 10	IL10	NM_000572.3	1.99	1.265	2.035	0.443
Hypocretin receptor type 2	HCRTR2	NM_001384272.1	2.685	–	3.695	–
Heme oxygenase 1	HMOX1	NM_002133.3	5.644	3.594	7.271	7.321
C-X-C motif chemokine ligand 6	CXCL6	NM_002993.4	2.022	1.949	2.308	0.335
C-reactive protein	CRP	NM_001329057.2	4.691	2.102	4.359	1.635
Cysteinyl leukotriene receptor 1	CYSLTR1	NM_001282187.2	2.195	1.094	3.877	0.341
Leptin	LEP	NM_000230.3	2.156	0.585	2.776	0.971
Corticotropin-releasing hormone receptor 2	CRHR2	NM_001883.5	Mental depression	2.383	1.769	3.004	1.349
Apolipoprotein A-IV	APOA4	NM_000482.4	4.195	2.689	3.504	0.988
Interleukin 5	IL5	NM_000879.3	2.325	1.132	3.277	1.79
C-reactive protein	CRP	NM_001329057.2	4.691	2.102	4.359	1.635
Interleukin 10	IL10	NM_000572.3	1.99	1.265	2.035	0.443
Cholinergic receptor nicotinic β 3 subunit	CHRNB3	NM_004198.3	3.294	-	2.606	-
Corticotrophin-releasing hormone	CRH	NM_000756.4	1.739	1.43	3.006	1.288
Interleukin 3	IL3	NM_000588.4	1.979	1.241	3.121	1.561
Dopamine receptor D5	DRD5	NM_000798.5	2.137	2.694	2.867	0.734
5-hydroxytryptamine	HTR1A	NM_000524.4	2.661	1.904	2.421	1.288
Gamma-aminobutyric acid receptor subunit α-5	GABRA5	NM_000810.4	1.593	1.192	2.104	0.291
Cytochrome P450 family 2 subfamily B member 6	CYP2B6	NM_000767.5	1.677	1.129	2.152	-
Heat shock protein family A	HSPA1A	NM_005345.6	4.756	2.851	6.42	5.097
Heat shock protein family A 1 like	HSPA1L	NM_005527.4	1.513	0.919	2.044	1.949

Disease gene analysis revealed downregulation of HCRTR2, nitric oxide synthase 1 (NOS1), statherin (STATH), CSF2, fibroblast growth factor 14 (FGF14), retrotransposon-like protein 1 (RTL1), pancreatic polypeptide (PPY), and H4 clustered histone 3 (H4C3) genes which are responsible for migraine disorder, cognition and mild cognitive disorders, depressive symptoms, neuralgia, and peripheral neuropathy in 100_9h vs. 100_3h (Figure 4F). Furthermore, genes related to mood and mental disorders, *viz*. chromogranin B (CHGB), dopamine beta-hydroxylase (DBH), PROKR2, vascular endothelial growth factor-D (VEGFD), and contactin-associated protein family member 4 (CNTNAP4), were observed to be downregulated in 50_9h vs. C_9h (Table 3).

The genes such as bradykinin receptor 1 (BDKRB1), NPY2R, PROKR2, SOX2 (sex determining region Y-box 2), Dickkopf-1 (DKK1), goosecoid homeobox (GSC), myeloid ecotropic insertion site 1 (MEIS1), and transcription factor AP-2β (TFAP2B) which were associated with unipolar depression, Alzheimer’s disease, sleep disturbances, and malignant glioma were downregulated in treated 100_3h vs. C_3h (Figure 4C). Genes contributing to Parkinsonian disorders such as GDNF family receptor alpha 2 (GFRA2), ATP-binding cassette subfamily C member 8 (ABCC8), potassium inwardly rectifying channel, subfamily J (KCNJ), synuclein alpha interacting protein (SNCAIP); chronic schizophrenia, chromogranin B (CHGB), DBH, NOS1, H4C (H4 clustered histone) family, netrin G1 (NTNG1); transient ischemic attack genes argininosuccinate lyase (ASL), NOS1, ADAMTS1, mental retardation genes ASCL1 (achaete-scute homolog 1), H4C family; panic disorders genes BDKRB family, NOS1; brain ischemia DBH, NOS1, CXCL12, ADAMTS1; sleep disorders and disturbances genes DBH, KCNJ family, SNCAIP, and PROKR2 were downregulated in 100_9h vs. C_9h (Figure 4D).

Disease ontology analysis revealed the downregulation of genes related to cerebral malaria, brain edema, Parkinson’s, cerebral infarction, multiple sclerosis, brain ischemia, and demyelinating disease (Figures 5A–H).

### 3.5 Dose and temporal comparisons

Disease ontology and DisGeNET analyses on WS-treated neuroblastoma cells were performed at different concentrations and time points. Dose comparison analysis revealed upregulated genes associated with brain disorders *viz.* alcohol dependence, brain ischemia, neuropathy, substance dependence, and post-traumatic stress disorder in 100_3h vs. 50_3h (Figure 5E). Moreover, dose comparison at 100_9h vs. 50_9h showed modulation of genes associated with brain edema, Parkinsonism, brain ischemia, visual epilepsy, brain and cerebral infarction, and paranoid schizophrenia (Figure 5F).

Furthermore, a time point comparison of higher WS treatment, i.e., 100_9h vs. 100_3h, showed downregulation of genes related to brain disorders such as migraine, autonomic neuropathy, movement disease, and Parkinson’s as well as upregulation of brain edema and middle cerebral artery infarction-related genes (Figure 5G). Interestingly, WS-treated neuroblastoma cells at 50_9h vs. 50_3h reported downregulation of cerebral malaria, brain edema, Parkinsonism, brain ischemia, synucleinopathy, neuropathy, multiple sclerosis, and sciatic neuropathy-related genes as well as upregulation of genes associated with cerebral palsy, cerebrovascular disease, demyelinating disease, narcolepsy, and meningitis (Figure 5H).

### 3.6 Real-time quantitative PCR expression analysis

**Figure 6:**
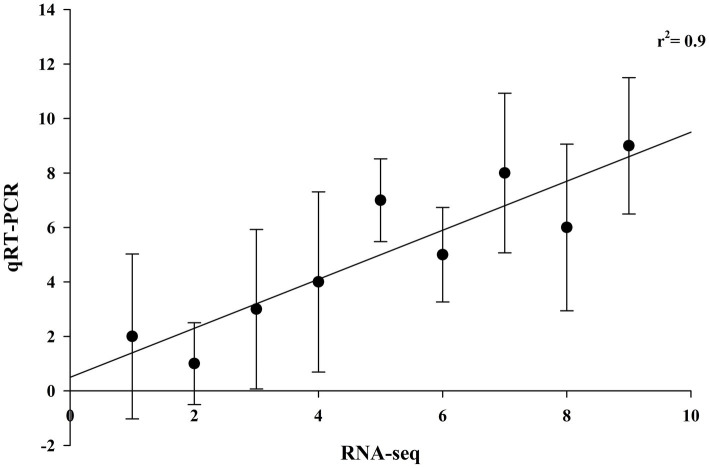
Validation of RNA-sequencing data with qRT-PCR data of brain-related disorders genes. Fold changes in gene expression measured by qRT-PCR for each sample in triplicate (*n* = 3) were plotted against fold change in sequencing data (coefficient of determination, r^2^ = 0.9).

To validate the RNA-seq data, the qRT-PCR expression pattern of genes representing major pathways having differential expression was correlated with RNA sequencing expression data with a coefficient of determination, r^2^ = 0.9, showing a good correlation between qRT-PCR and RNA-sequencing data (Figure 6).

### 3.7 Western blotting analysis

**Figure 7:**
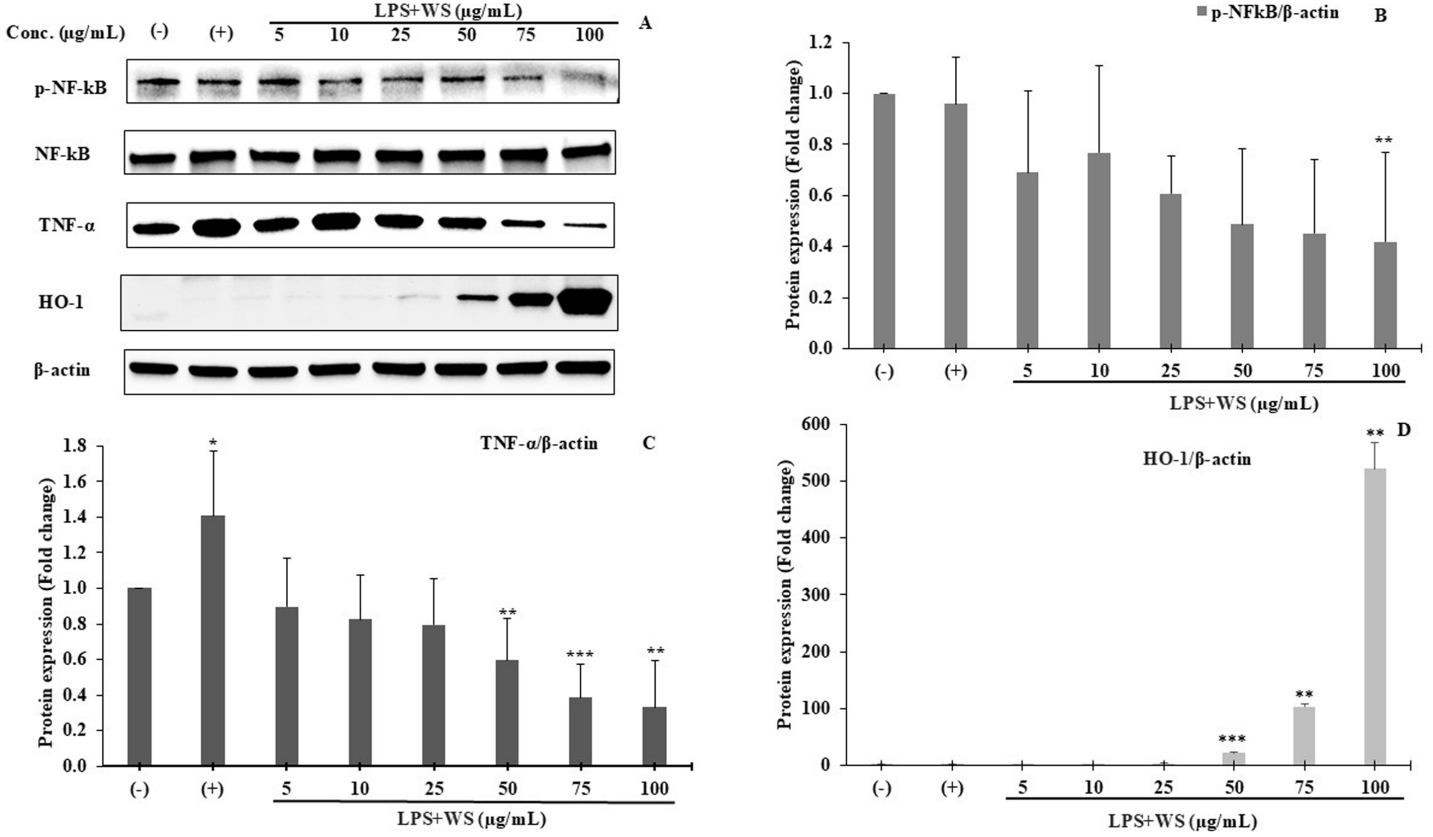
**(A)** Western blot analysis of p-NF-κB, NF-κB, TNF-*α*, and HO-1. Representative of three independent experiments. **(B)** Densitometric analysis of p-NF-κB expression. All data are represented as means ± SD (**p < 0.01 as compared to the control). **(C)** Densitometric analysis of TNF-α expression. All data are represented as means ± SD (*p < 0.05, **p < 0.01, and ***p < 0.005 as compared to the control). **(D)** Densitometric analysis of HO-1 expression. All data are represented as means ± SD (**p < 0.01 and ***p < 0.005 as compared to the control). (−) represents cells treated with DMSO; (+) represents cells treated with DMSO and LPS.

The expressions of phosphorylated NF-κB (p-NF-κB), pro-inflammatory cytokines (TNF-*α*), and HO-1 were examined using Western blot analysis. LPS-stimulated SK-N-SH cells showed an increase in the expressions of p-NF-κB and TNF-α compared to normal control SK-N-SH cells (Figure 7A) (*p* ≤ 0.05). In addition, NF-κB phosphorylation decreased significantly in LPS-induced SK-N-SH cells treated with 100 μg/mL WS compared to the LPS-stimulated control group (Figure 7B) (p ≤ 0.05). Furthermore, the expression of TNF-α showed enhanced protein expressions in LPS-induced SK-N-SH cells in comparison with the normal control group (Figure 7C) (*p* ≤ 0.05). However, a significant decrease in protein expressions of TNF-α at 50, 75, and 100 μg/mL in WS-treated cells compared to the LPS-stimulated control group was observed (Figure 7C) (*p* ≤ 0.05). The expression of the antioxidant enzyme HO-1 was significantly upregulated in WS-treated cells compared to the LPS-induced group (Figure 7D). Moreover, upregulation of HO-1 was found to be significant at higher concentrations of WS, i.e., 50 (*p* < 0.005), 75 (*p* < 0.01), and 100 (p < 0.01) μg/mL in comparison with LPS-treated cells (Figure 7D).

## 4 Discussion

Over 10 million people worldwide suffer from neurological illnesses, and the number is projected to rise in the coming years. Approximately 3.1% of people in the West aged between 70 and 79 years are more susceptible to neurodegenerative disorders compared to 0.1% of a similar age group in India (Makkar et al., 2020). Phytochemical constituents from plant sources have excellent therapeutic properties, thus creating new opportunities for research in nutrition and health. Nutrients and herbal remedies work wonders for illnesses linked to lifestyle, including mental health (Dohrmann et al., 2019). WS has been well known for its health attributes, and extensive studies have been conducted to define the biological activity of WS (Basudkar et al., 2024; Saha et al., 2024) and a growing body of evidence has been accumulated. However, a comprehensive definition of the whole-genome transcriptome and changes modulated therein by WS administration have not been explored thus far. Hence, a whole-genome sequencing study of WS on neuroblastoma SK-N-SH cells was performed to understand the key mechanism of action involved especially in brain-related disorders.

Oxidative stress triggers the generation of different free radicals which consequently cause damage to the machinery of the cell. Cells adopt an antioxidant defense strategy in which free radical is converted to H_2_O_2_ and O_2_ which is further scavenged to water and oxygen (Olufunmilayo et al., 2023). Upregulated expression of antioxidant genes, *viz*., HMOX1, NFE2l2, SLC7A11, and NADPH oxidase, and downregulated expression of monooxygenase enzymes in our study could be a putative approach to deal with the oxidative stress in WS-treated human neuroblastoma SK-N-SH cells. The upregulation of NFE2l2 clearly depicts the central role of NRF2 in reducing oxidative stress, a key marker of numerous neurodegenerative diseases (Levings et al., 2023).

WS root extract constitutes withaferin A, withanolides, and withanosides, and at the molecular level, these compounds show beneficial effects in neurodegenerative diseases *via* inhibition of NF-κB activation, preservation of synaptic function, effect on cholinergic markers reduction, and improvement in antioxidant effects which is mediated by the migration of NRF2 to the nucleus, thereby resulting in the activation of antioxidant enzymes (Das et al., 2021). We have previously reported the regulatory potential for crosstalk between Nfkb1- and Nrf2-mediated gene expression in cancer and inflammation (Nair et al., 2008). Moreover, withaferin A effectively inhibits the NF-κB activation by interrupting phosphorylation and deterioration through suppression of IκB kinase stimulation which in turn suppresses the transcription of various pro-inflammatory cytokines and NO-synthase (NOS) which is further confirmed using Western blotting. Withaferin A and other compounds in WS inhibit inflammatory diseases in various *in vitro* and *in vivo* models of neurodegenerative disorders by mediating critical inflammatory pathways such as NF-κB and NRF2 signaling (Logie and Vanden Berghe, 2020). In addition, the NFE2L2-ARE pathway is stimulated within a cell to generate antioxidants to combat oxidative stress.

NOS1, which mediates the production of NO which plays a crucial role in schizophrenia and cognitive function, was found to be downregulated on treatment of neuroblastoma cells with WS. Previous evidence suggested the role of NOS1 in cognitive performance *via* its effect on prefrontal functioning (O’Donoghue et al., 2012; Zhang et al., 2015). At the synaptic machinery, nitrergic signaling, the NMDA synapse, and interactions between NO and dopamine in the striatum are critical in schizophrenia and possibly bipolar disorder. The link between NO and impulsive behaviors, depression, and anxiety might be due to serotonergic and nitrergic interactions. NO contribution to neurodegenerative disorders might be due to the involvement of increased oxidative stress and glutamate excitotoxicity. Therefore, NOS1 indirectly serves manifold functions, and its dysfunction contributes to many different pathologies (Freudenberg et al., 2015).

HMOX1 induction protects against dopamine neurotoxins by inducing the synthesis of neurotrophic factors originating from the brain and glial cells. Several lines of evidence highlighted the HMOX1 dysfunction related to brain inflammation and neurodegeneration, comprising Parkinson’s and Alzheimer’s diseases (Wu and Hsieh, 2022). SLC7A11 is a crucial biomarker in neurodegenerative diseases and brain cancers, and overexpression of SLC7A11 results in an imbalance in cellular glutamate homeostasis (Lee and Roh, 2022). Targeting SLC7A11 is an important selective biomarker for novel brain-associated disorders (Saito and Soga, 2021).

The downregulation of DKK1 and ADMATS1 plays a crucial role in Alzheimer’s disease. Recent studies have supported the role of increased expression of negative secretory protein DKK1 in the Wnt signaling pathway which was closely associated with the progression of neurodegenerative diseases *viz*. Alzheimer’s disease, brain ischemia, and temporal lobe epilepsy. DKK1 reduces the formation or synapses assembly *via* inhibition of canonical Wnt signaling pathway, effecting memory function, and thus causing neuronal apoptosis in the cell (Ren et al., 2019). DKK1 inhibits endogenous Wnt ligands which play a crucial role in the maintenance of neural synapses (Purro et al., 2014).

Parkinson’s disease is one the most prevalent neurodegenerative disorders affecting 6 million people and is expected to double in prevalence by 2040 (Dorsey et al., 2018). SNCA is considered as a major causative gene involved in onset of Parkinson’s disease. Oxidative stress in Parkinson’s enhances the aggregation of SNCA enhancing ER stress which in turn increases caspase-3, caspase-9, and caspase-12 activity. SNCA was found to be downregulated in WS-treated neuroblastoma cells pointing to the role of WS in SNCA downregulation, thus highlighting its role in the suppression of Parkinson’s disease (Siddiqui et al., 2016). The downregulation of SNCA in WS-treated cells could be *via* inhibition of the PI3K/AKT/mTOR pathway (Shen et al., 2017). However, further studies are needed to support this.

Interestingly, ATP-binding cassettes KCNJ and ABCC8 involved in neonatal and maturity onset of diabetes and PD have been observed to be downregulated on treatment with WS deciphering the neuroprotective and antidiabetic role of WS in SK-N-SH cells (Day and Mullin, 2021; Ivanoshchuk et al., 2023).

Depressive and mood disorders are characterized by debilitating forms of illness and globally cover 4.4% of the total population suffering from depression. Studies based on the antidepressant effect provide strong evidence of NPY system alterations in stress response, depression pathophysiology, and suicide (Heilig et al., 1988). The increased mRNA expression of NPY receptors, especially NPY2R, has been observed in depression subjects which is suggestive of aggravated anxiety (Sharma et al., 2022); however, the results in our study were not in consonance with the previous finding. However, specific alterations in the NPY system may be useful as potential biomarkers or predictors of suicide. The mechanism of action of NPY2R shows augmented cAMP signaling on WS treatment to SK-N-SH cells. Moreover, NPY induces PI3K/Akt/mTOR/eIL4E signaling pathway activation, which results in the production of transforming growth factor (TGF), leading to the upregulation of TGF-*β* expression (Zhang et al., 2021).

Recently, a potential gene that may affect a person’s risk of developing schizophrenia is CHGB, and its variants on schizophrenia might possibly affect the levels of dopamine *via* interfering with neurotransmission pathway (Shin et al., 2017). Recent studies highlighted the correlation between schizophrenia and dopamine. An *in vivo* study suggested enhanced concentrations of synaptic dopamine in the brain of patients with schizophrenia (Kegeles et al., 2010); however, another *in vitro* study revealed lowered CHGB levels in the schizophrenia patients’ cerebrospinal fluid (Landén et al., 1999). In our study, CHGB was downregulated by WS treatment. Further studies are needed to clearly highlight the correlation between dopamine and the role of the CHGB gene in schizophrenia.

Aquaporins are crucial in brain tumors, cerebral ischemia, bacterial meningitis, and other conditions. The expression of AQP4 is upregulated in astrocytes and mediates the formation of brain edema along with its elimination. Furthermore, AQP4 contributes to the solute’s clearance through a paravascular pathway from the interstitial fluid of normal brain (Papadopoulos and Saadoun, 2015). AQP4 was found to be upregulated in our study.

Super-enhancer-associated INSM2 was downregulated in WS-treated SK-N-SH cells in our study. INSM2 mediates the lipid metabolism SREBP1expression *via* the mTOR signaling pathway, which further interferes with lipid metabolism in neuroblastoma (Cao et al., 2022).

Although this is the first study on the modulation of gene expression by WS treatment involved in brain-related disorders, this study also has some limitations. First, this is an exploratory study, using SK-N-SH, a common cell line used for neurodegenerative diseases; however, to further validate our findings, differentiated neuronal cell lines, primary neuronal cultures, or 3D organoids should be used in future studies. Second, to enhance the relevance of our findings, *in vivo* models should be included in future research.

## 5 Conclusion and future perspectives

The present study is a whole-genome transcriptomic profiling of WS-treated human neuroblastoma cells by RNA-sequencing employing a dose and time-dependent approach. Our study effectively identified regulatory biomarkers modulating various neurodegenerative disorders *via* signaling molecules. Regulation of signaling pathways involved at the genomic level by WS was found to be important in modulating brain-related disorders. Interestingly, our sequencing study showed that WS could modulate several key genes that were implicated in Alzheimer’s disease, Parkinson’s disease, migraine, depression, bipolar disorder, forgetfulness, stress, anxiety, cognition, sleep disorders, and substance abuse among others. In addition, our data with WS provide valuable information about potential key biomarkers that may serve as targets for the prevention and treatment of neurodegenerative diseases. To the best of our knowledge, our study reveals for the first time that WS may modulate key genes in neurodegenerative disorders. This may have important beneficial implications for protective interventions with WS for brain-related disorders and healthy aging. Furthermore, future research needs to emphasize the experimental validation of key pathways *via* quantitative analysis of key protein of pathway components *viz.* NRF2 and HMOX1 along with assessment of their functional roles in oxidative stress and neuroinflammation. Moreover, functional assays including measurement of reactive oxygen species (ROS), mitochondrial activity, and apoptosis markers should be performed to demonstrate the biological relevance of the findings. Furthermore, to confirm the physiological relevance of WS effects, primary neuronal cultures or differentiated SH-SY5Y cells should be used to evaluate synaptic protein levels, such as synaptophysin or PSD-95, and assessment of neuronal viability after WS treatment would provide critical insights into its neuroprotective potential.

## Data Availability

The datasets presented in the study can be found in online repositories. The names of the repository/repositories and accession number(s) are as follows: https://www.ncbi.nlm.nih.gov/, SRA repository, BioProject accession number PRJNA1168412.
