# Supplementary material for: Whole-genome sequencing shows modulation of neurodegenerative genes by Withania somnifera in human SK-N-SH cells
Source: Front Mol Neurosci. 2025 Jun 25;18:1512727. doi: 10.3389/fnmol.2025.1512727 (PMC12238756; doi:10.3389/fnmol.2025.1512727)
Supplement: Supplementary file 2 [file Supplementary_file_2.docx]

Supplementary Material

# Supplementary Figures and Tables

## Supplementary Figures

**Figure S1:** Reactome pathway (50 μg/mL_3h *vs* C_3h).


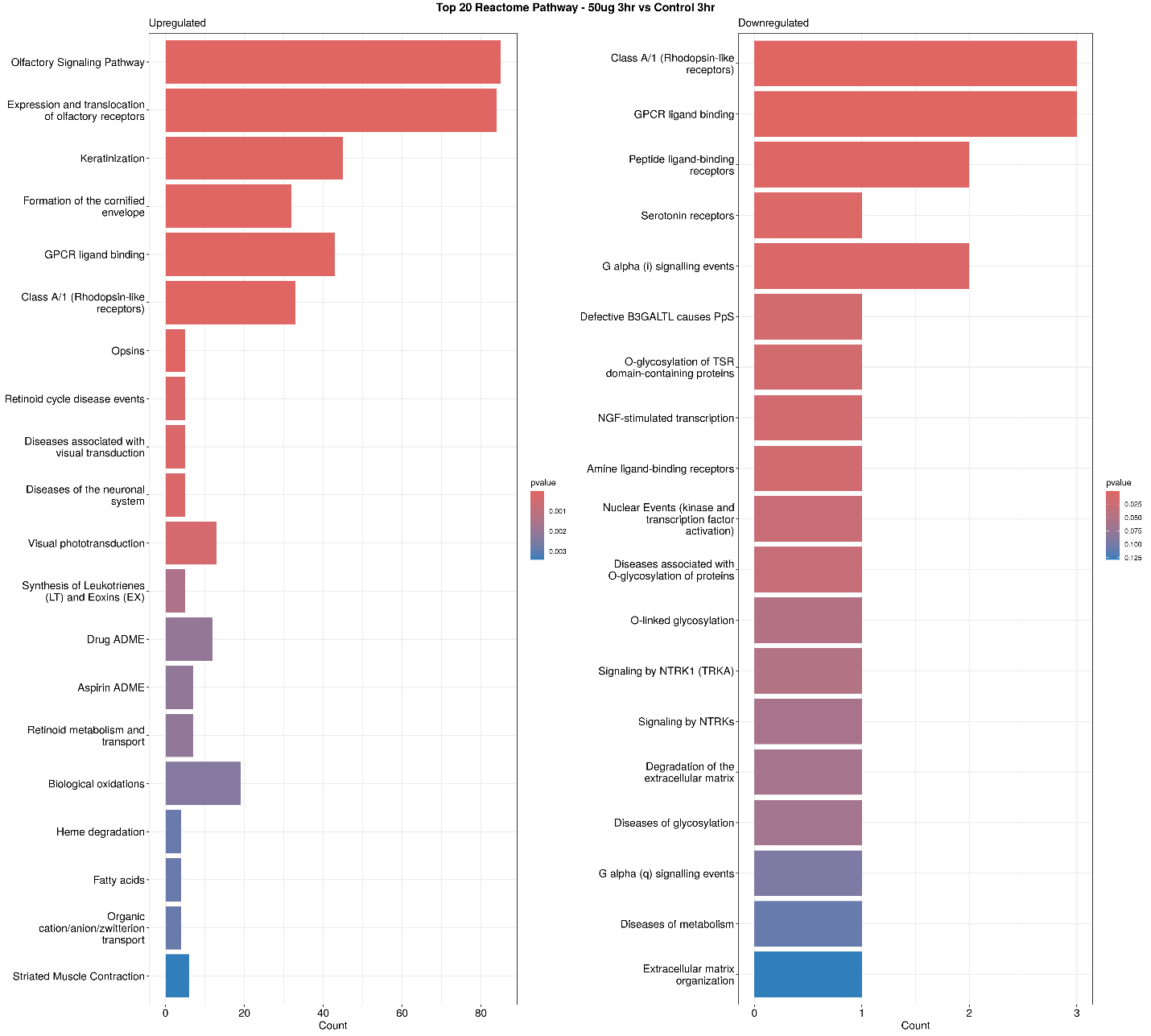


**Figure S2:** Reactome pathway (50 μg/mL_9h *vs* C_9h).


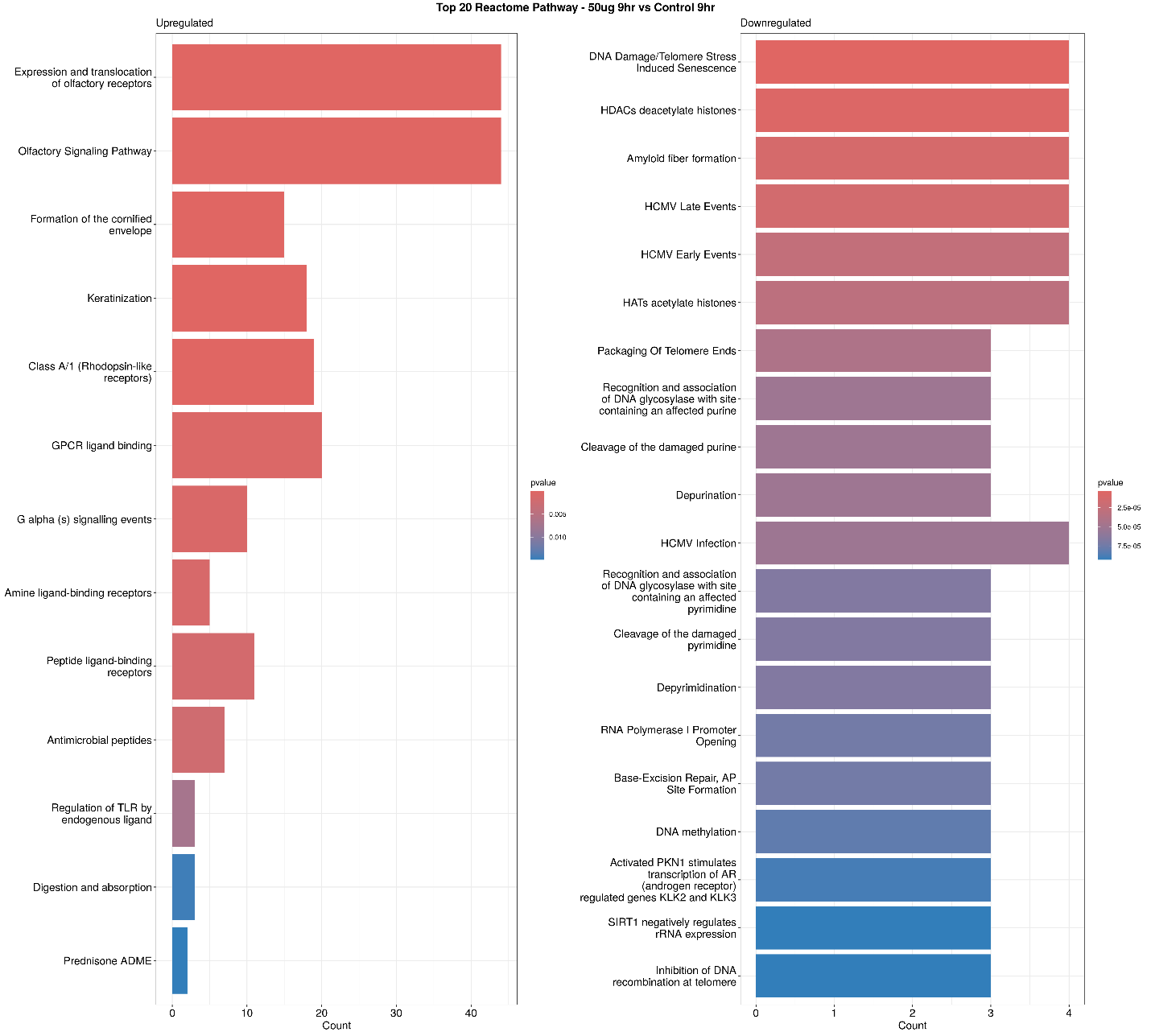


**Figure S3:** Reactome pathway (100 μg/mL_3h *vs* C_3h).


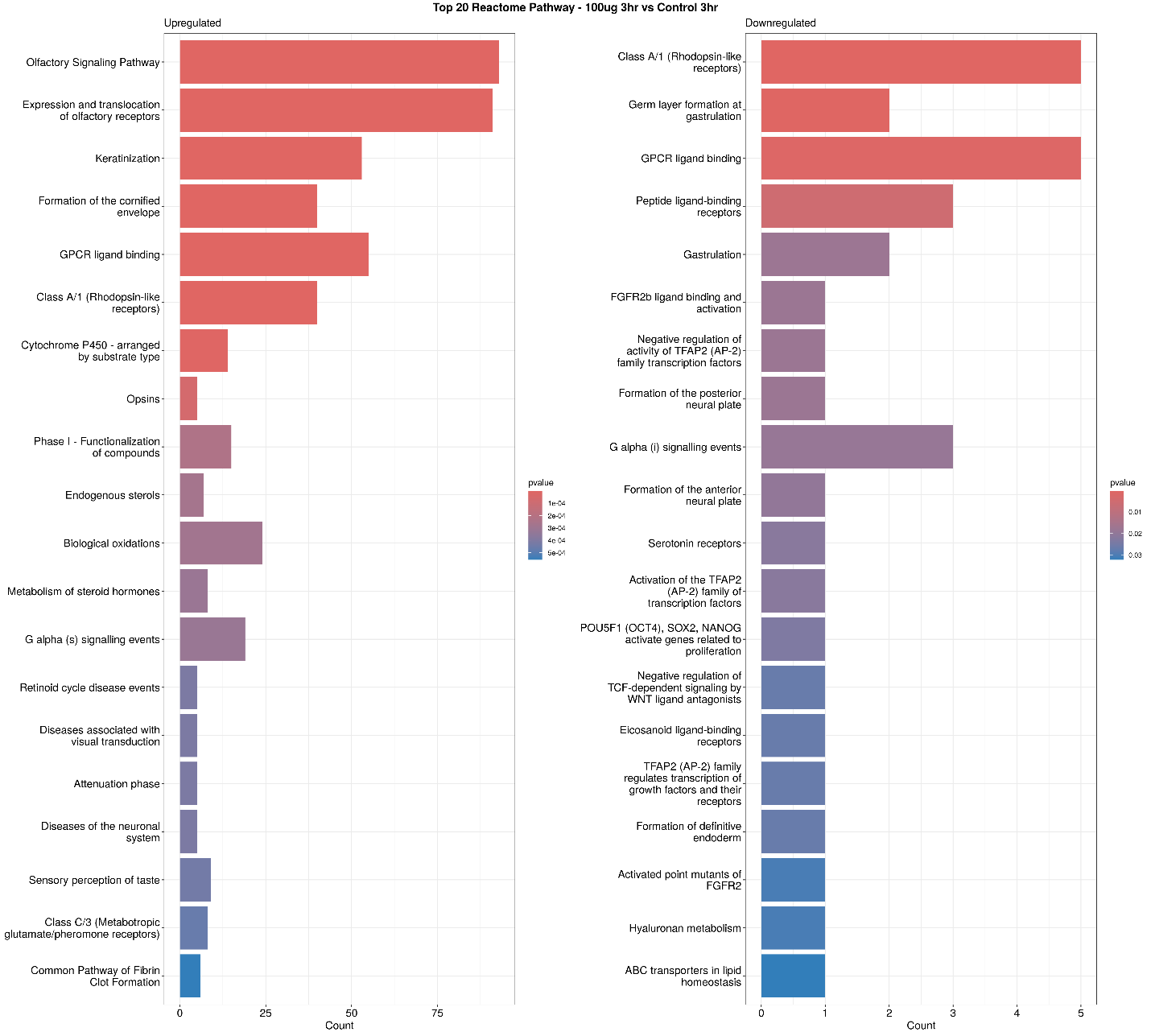


**Figure S4**: GO Dose comparison; Reactome pathway (100 μg/mL_3h *vs* 50 μg/mL_3h).


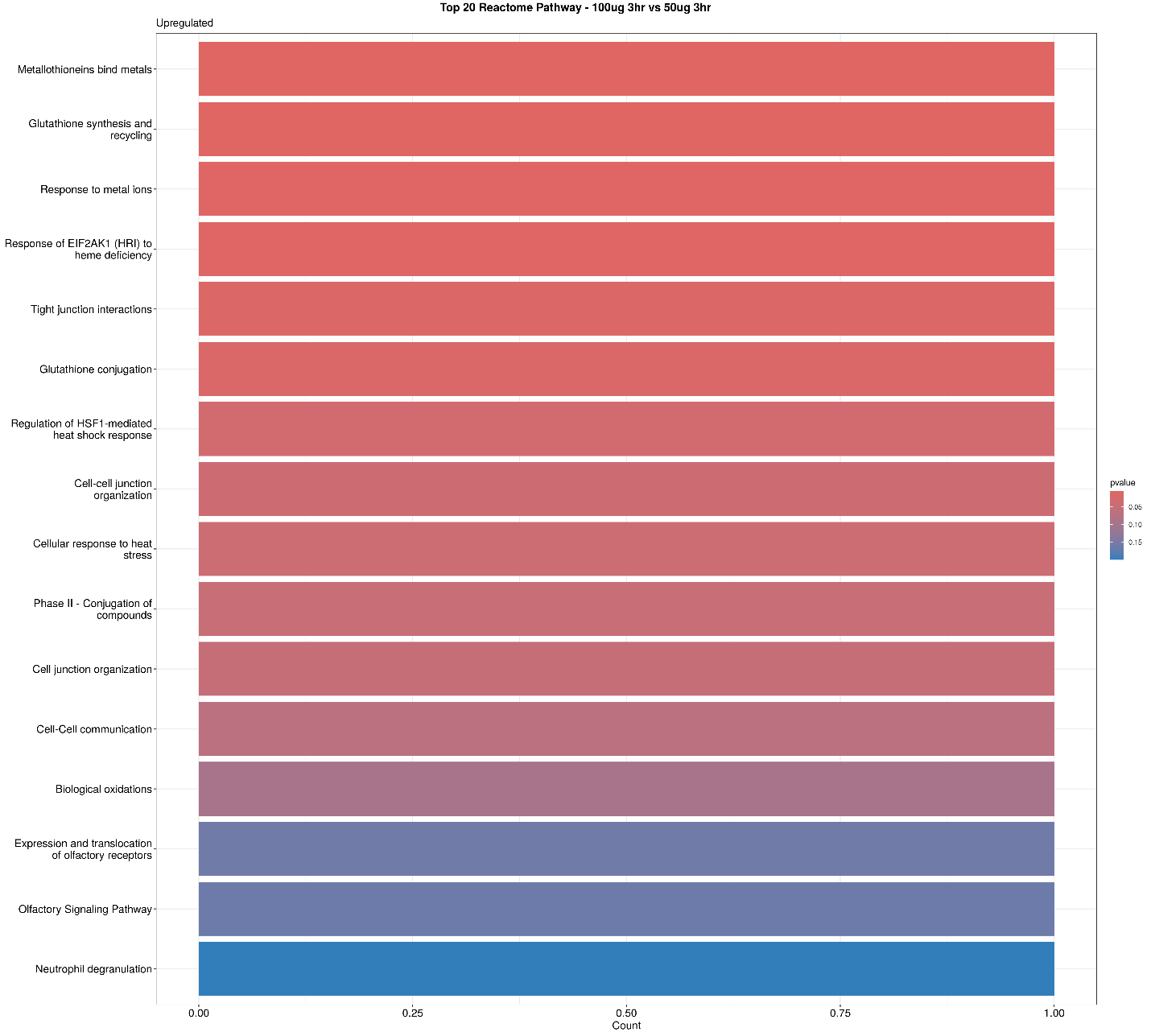


**Figure S5**: GO Dose comparison; Reactome pathway (100 μg/mL_9h *vs* 50 μg/mL_9h).


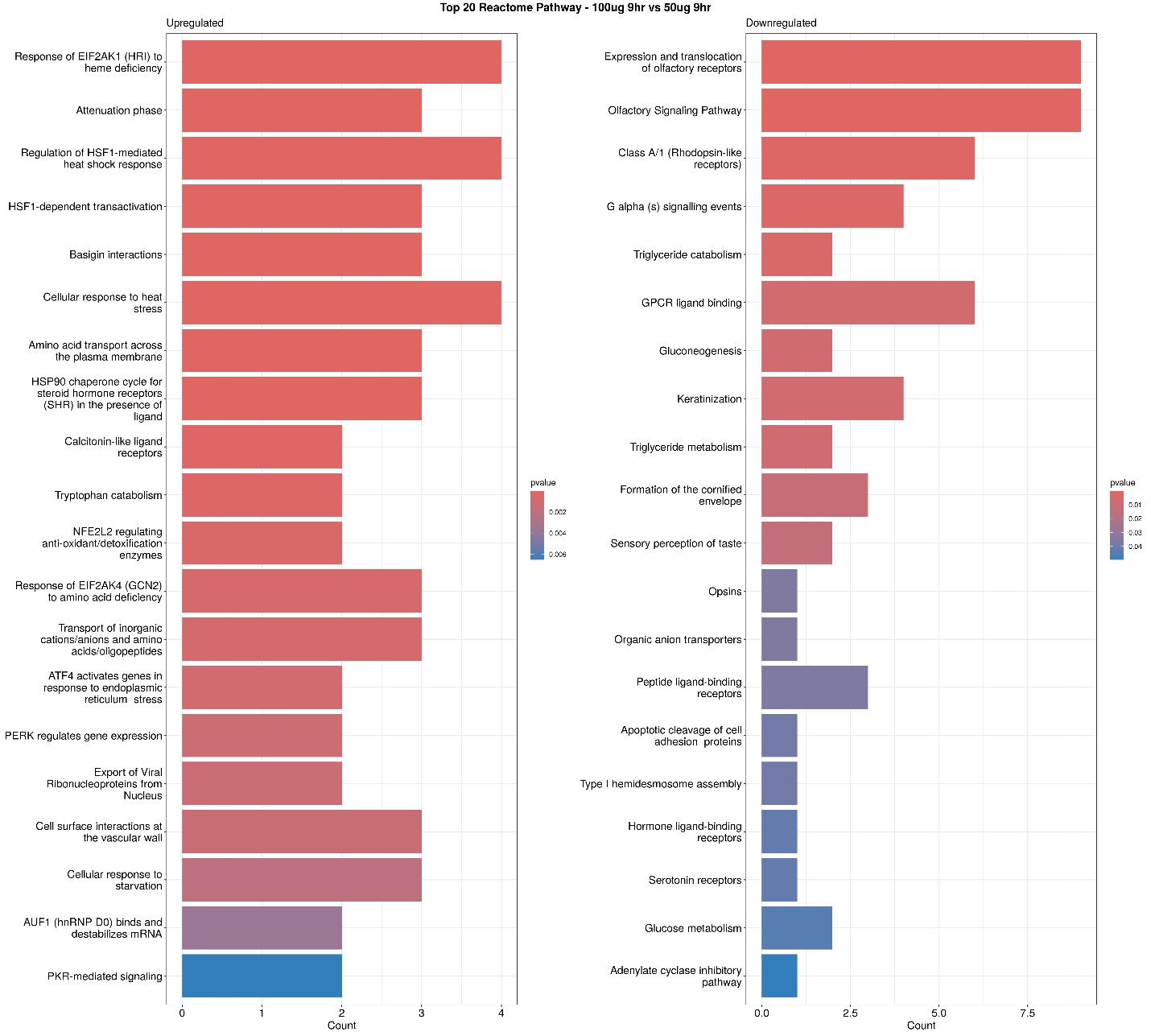


**Figure S6**: GO Time comparison; Reactome pathway (50 μg/mL_9h *vs* 50 μg/mL_3h).


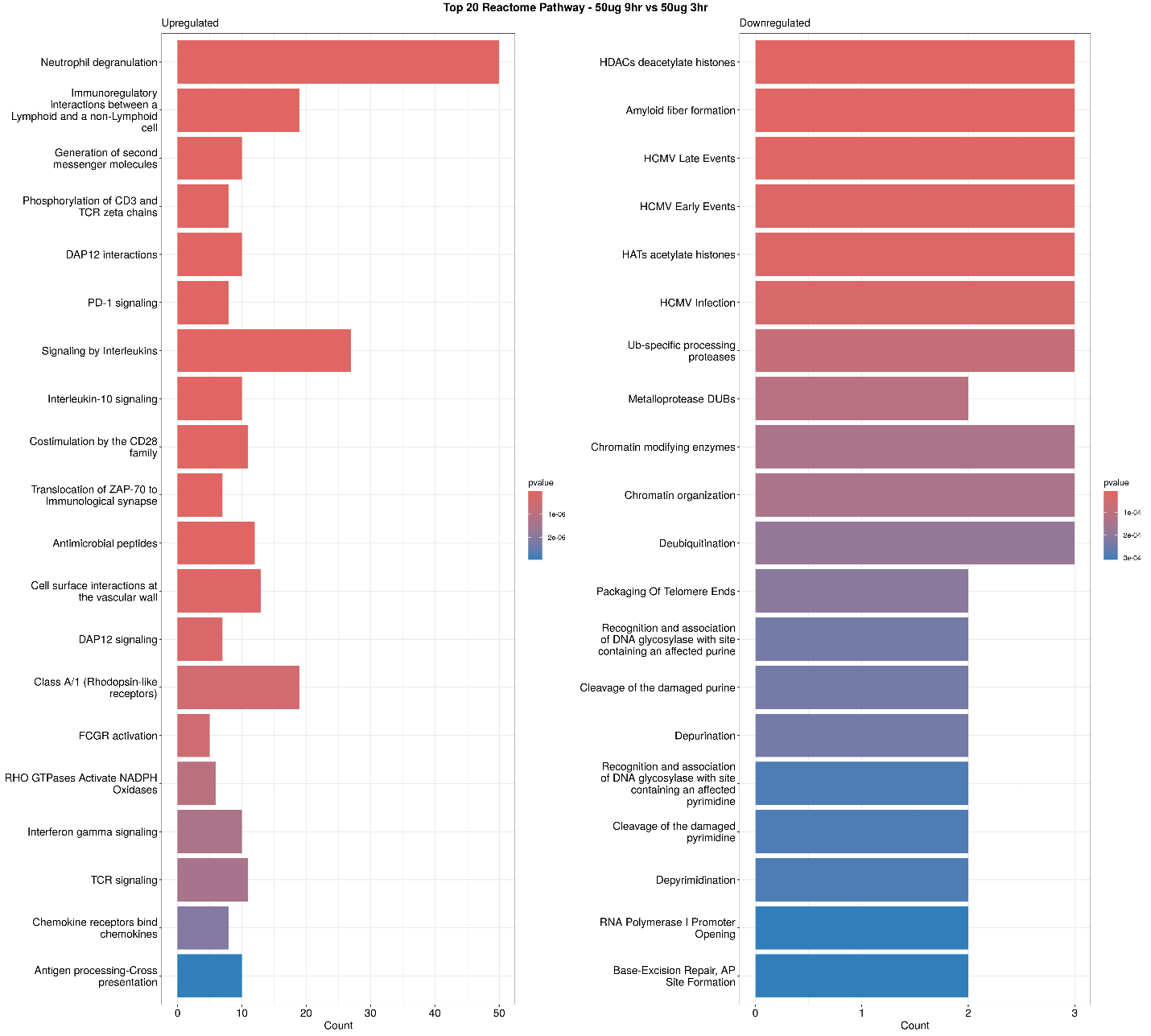


**Figure S7**: GO Time comparison; Reactome pathway (100 μg/mL_9h *vs* 100 μg/mL_3h).


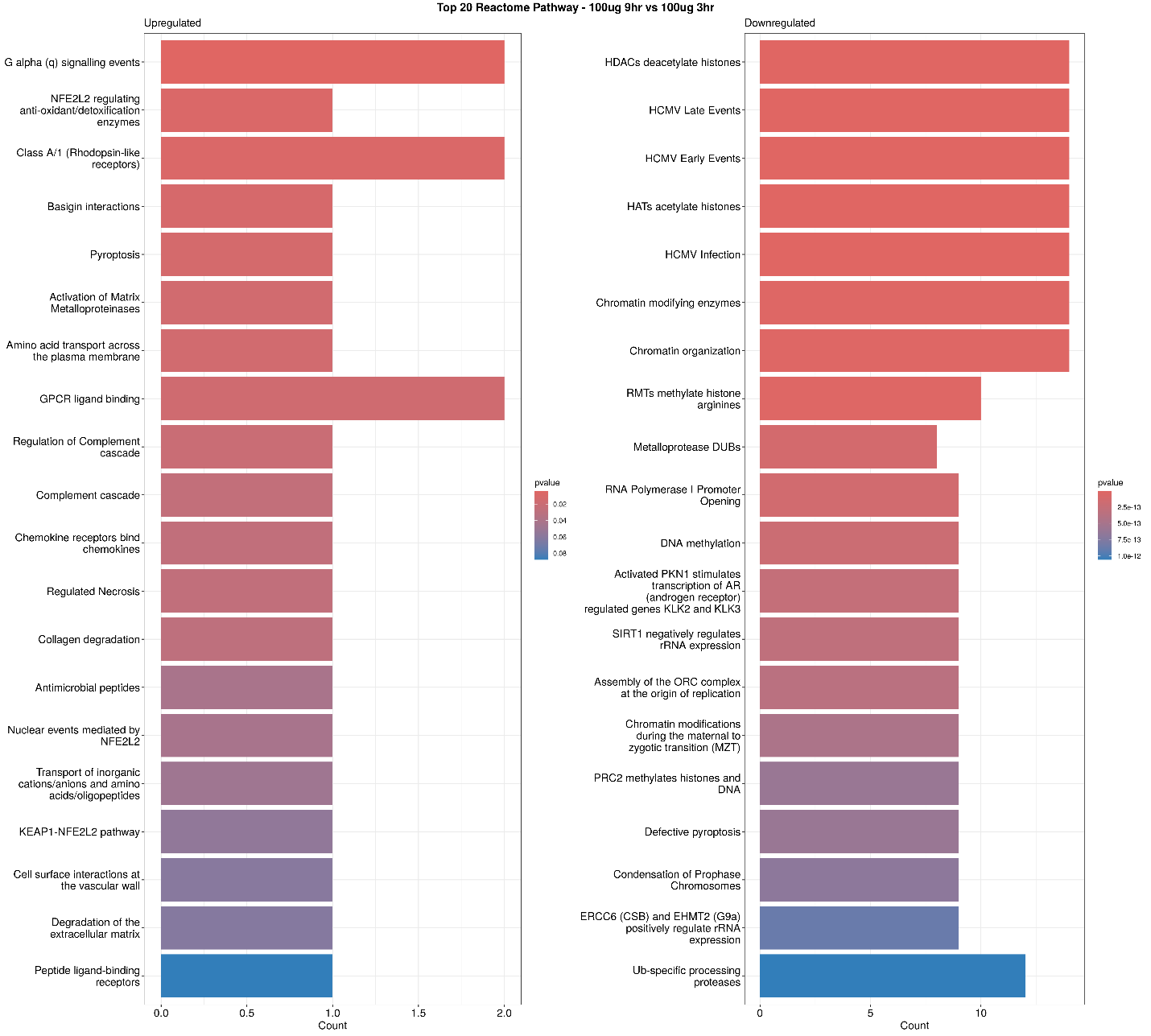


**1.2 Supplementary Tables**

**Table S2**. Disease ontology analysis of 100 μg/mL_3h *vs* 50 μg/mL_3h WS-treated SK-N-SH cells.

| **Description** | **p-value** | **Gene ID** |
| --- | --- | --- |
| **Upregulated** |  |  |
| **Other diseases** |  |  |
| Ureteral obstruction | 0.02781 | CASP12 |
| Urinary tract obstruction | 0.03302 | CASP12 |
| Endocrine organ benign neoplasm | 0.03349 | CLDN8 |
| Substance dependence | 0.04149 | GGTLC1 |

**Table S3**. Disease ontology analysis of 100 μg/mL_9h *vs* 50 μg/mL_9h WS-treated SK-N-SH cells.

| **Description** | **p-value** | **Gene ID** |
| --- | --- | --- |
| **Upregulated** |  |  |
| **Other diseases** |  |  |
| Tongue squamous cell carcinoma | 1.342E-05 | SLC7A5/SLC7A11/SLC3A2 |
| Essential hypertension | 4.794E-05 | HMOX1/GDF15/HSPA1B/HSPA1A |
| Pulmonary venoocclusive disease | 0.00010 | HMOX1/DDIT3 |
| Oropharynx cancer | 0.00015 | SLC7A5/SLC3A2 |
| Congestive heart failure | 0.00015 | HMOX1/CALCA/GDF15/HSPA1B/HSPA1A |
| Bacterial infectious disease | 0.00016 | HMOX1/CALCA/ATF3/DDIT3/HSPA1B |
| Sarcomatoid carcinoma | 0.00020 | SLC7A5/SLC3A2 |
| Intestinal obstruction | 0.00023 | HMOX1/CALCA |
| Pharynx cancer | 0.00040 | SLC7A5/SLC3A2/HSPA1B |
| Acute kidney failure | 0.00060 | HMOX1/DDIT3/HSPA1A |
| Hemochromatosis | 0.00096 | HMOX1/GDF15 |
| Asthma | 0.00143 | HMOX1/CALCA/HSPA1B/HSPA1A |
| Bronchial disease | 0.00161 | HMOX1/CALCA/HSPA1B/HSPA1A |
| Toxic shock syndrome | 0.00215 | HMOX1/HSPA1B |
| Atopic dermatitis | 0.00260 | HMOX1/CALCA/ADM2 |
| Allergic contact dermatitis | 0.00298 | HMOX1/CALCA/ADM2 |
| Contact dermatitis | 0.00311 | HMOX1/CALCA/ADM2 |
| Tuberculosis | 0.00339 | CALCA/ATF3/DDIT3 |
| Upper respiratory tract disease | 0.00344 | HMOX1/CALCA/HSPA1A |
| Acute pancreatitis | 0.00355 | CALCA/HSPA1B |
| Head and neck squamous cell carcinoma | 0.00373 | SLC7A5/SLC7A11/SLC3A2 |
| Kawasaki disease | 0.00457 | HMOX1/HSPA1A |
| Lymphadenitis | 0.00513 | HMOX1/HSPA1A |
| Lymph node disease | 0.00513 | HMOX1/HSPA1A |
| Cell type benign neoplasm | 0.00540 | HMOX1/SLC7A5/GDF15/SLC3A2 |
| Osteoarthritis | 0.00577 | HMOX1/TRIB3/CALCA |
| Dermatitis | 0.00610 | HMOX1/CALCA/ADM2 |
| Metal metabolism disorder | 0.00649 | HMOX1/GDF15 |
| Commensal bacterial infectious disease | 0.00681 | HMOX1/HSPA1B |
| Lung adenocarcinoma | 0.00718 | HMOX1/SLC7A5/SLC3A2 |
| Transitional cell carcinoma | 0.00748 | SLC7A5/SLC3A2 |
| Head and neck carcinoma | 0.00764 | SLC7A5/SLC7A11/SLC3A2 |
| Pulmonary tuberculosis | 0.00765 | ATF3/DDIT3 |
| Head and neck cancer | 0.00820 | SLC7A5/SLC7A11/SLC3A2 |
| Peptic ulcer disease | 0.00984 | HMOX1/HSPA1A |
| Primary bacterial infectious disease | 0.00984 | CALCA/ATF3/DDIT3 |
| Respiratory failure | 0.01062 | HMOX1/HSPA1A |
| Pre-eclampsia | 0.01117 | HMOX1/CALCA/GDF15 |
| Acute myocardial infarction | 0.01123 | HMOX1/CALCA |
| Allergic rhinitis | 0.01381 | HMOX1/CALCA |
| Gastric adenocarcinoma | 0.01426 | HMOX1/SLC7A5 |
| Leiomyoma | 0.01449 | SLC7A5/SLC3A2 |
| Lymphatic system disease | 0.01472 | HMOX1/HSPA1A |
| Status epilepticus | 0.01518 | HMOX1/HSPA1A |
| Extrahepatic bile duct carcinoma | 0.01541 | SLC7A5 |
| Systolic heart failure | 0.01541 | HSPA1A |
| Middle cerebral artery infarction | 0.01613 | HMOX1/ARC |
| Kidney failure | 0.01685 | HMOX1/DDIT3/HSPA1A |
| Hepatopulmonary syndrome | 0.01694 | HMOX1 |
| Atherosclerosis | 0.01735 | HMOX1/TRIB3/HSPA1A |
| Arteriosclerotic cardiovascular disease | 0.01748 | HMOX1/TRIB3/HSPA1A |
| Visual epilepsy | 0.01760 | ARC/HSPA1A |
| Adult-onset Still's disease | 0.01847 | HMOX1 |
| Lung large cell carcinoma | 0.01847 | SLC7A5 |
| Autosomal dominant limb-girdle muscular dystrophy | 0.02000 | DNAJB1 |
| Paranoid schizophrenia | 0.02000 | HSPA1A |
| Obstructive jaundice | 0.02000 | HMOX1 |
| Pituitary cancer | 0.02000 | CALCA |
| Alcoholic pancreatitis | 0.02000 | HSPA1B |
| Herpes simplex | 0.02000 | DNAJB1 |
| Liposarcoma | 0.02152 | DDIT3 |
| Meniere's disease | 0.02152 | HSPA1A |
| Colon cancer | 0.02271 | HMOX1/SLC7A5/HSPA1A |
| Platelet storage pool deficiency | 0.02304 | SLC7A11 |
| Senile cataract | 0.02304 | HSPA1B |
| Peripheral vertigo | 0.02304 | HSPA1A |
| Endolymphatic hydrops | 0.02304 | HSPA1A |
| Rhinitis | 0.02375 | HMOX1/CALCA |
| Arteriosclerosis | 0.02391 | HMOX1/TRIB3/HSPA1A |
| Nasal cavity disease | 0.02403 | HMOX1/CALCA |
| Hypercalcemia | 0.02456 | CALCA |
| Intestinal disease | 0.02483 | HMOX1/CALCA/HSPA1B |
| Pneumonia | 0.02607 | HMOX1/CALCA |
| Vestibular disease | 0.02607 | HSPA1A |
| Visceral leishmaniasis | 0.02607 | HMOX1 |
| Nose disease | 0.02726 | HMOX1/CALCA |
| Varicocele | 0.02759 | HMOX1 |
| Pelvic varices | 0.02759 | HMOX1 |
| Colitis | 0.02847 | HMOX1/HSPA1B |
| Prediabetes syndrome | 0.02910 | CALCA |
| Prostatitis | 0.02910 | HSPA1A |
| Hepatic encephalopathy | 0.03061 | HMOX1 |
| Coronary restenosis | 0.03061 | HMOX1 |
| Myocardial infarction | 0.03079 | HMOX1/CALCA/GDF15 |
| Movement disease | 0.03129 | HMOX1/HSPA1A |
| Cystic fibrosis | 0.03193 | HMOX1/HSPA1A |
| Pulmonary edema | 0.03211 | HSPA1A |
| Factor VIII deficiency | 0.03211 | HMOX1 |
| Borna disease | 0.03211 | DDIT3 |
| Pancreatitis | 0.03257 | CALCA/HSPA1B |
| Systemic Lupus erythematosus | 0.03487 | CALCA/HSPA1A |
| Pancreas disease | 0.03621 | CALCA/HSPA1B |
| Lupus erythematosus | 0.03689 | CALCA/HSPA1A |
| Cerebral infarction | 0.03757 | HMOX1/ARC |
| Common cold | 0.03812 | HSPA1A |
| Stomach carcinoma | 0.03826 | HMOX1/SLC7A5 |
| Hypospadias | 0.03962 | ATF3 |
| Pyelonephritis | 0.03962 | CALCA |
| Pyelitis | 0.03962 | CALCA |
| Cerebral malaria | 0.04111 | HMOX1 |
| Hermansky-Pudlak syndrome | 0.04260 | SLC7A11 |
| Varicose veins | 0.04260 | HMOX1 |
| Colon carcinoma | 0.04394 | HMOX1/HSPA1A |
| Pulmonary hypertension | 0.04394 | HMOX1/DDIT3 |
| Rectum cancer | 0.04409 | SLC7A5 |
| Hyperhomocysteinemia | 0.04409 | ATF3 |
| Hepatitis B | 0.04615 | HMOX1/DNAJB1 |
| Henoch-Schoenlein purpura | 0.04706 | HMOX1 |
| Renovascular hypertension | 0.04706 | HMOX1 |
| Leishmaniasis | 0.04855 | HMOX1 |
| Hypersensitivity vasculitis | 0.04855 | HMOX1 |

**Table S4.** Disease ontology analysis of 100 μg/mL_9h *vs* 100 μg/mL_3h WS-treated SK-N-SH cells.

| **Description** | **p-value** | **Gene ID** |
| --- | --- | --- |
| **Upregulated** |  |  |
| **Other diseases** |  |  |
| Leukopenia | 0.00028 | XCL1/ELANE |
| Leukocyte disease | 0.00081 | XCL1/ELANE |
| Blood coagulation disease | 0.00301 | SLC7A11/ELANE |
| Severe congenital neutropenia | 0.00387 | ELANE |
| Periapical periodontitis | 0.00387 | ELANE |
| Platelet storage pool deficiency | 0.00581 | SLC7A11 |
| Essential thrombocythemia | 0.00696 | ELANE |
| Diffuse scleroderma | 0.00774 | XCL1 |
| Lymphopenia | 0.00812 | XCL1 |
| Disseminated intravascular coagulation | 0.00851 | ELANE |
| Pleurisy | 0.01043 | ELANE |
| Granulomatosis with polyangiitis | 0.01043 | XCL1 |
| Hermansky-Pudlak syndrome | 0.01082 | SLC7A11 |
| Tongue squamous cell carcinoma | 0.01197 | SLC7A11 |
| Myeloproliferative neoplasm | 0.01274 | ELANE |
| Peritonitis | 0.01313 | ELANE |
| Pleural disease | 0.01696 | ELANE |
| Toxic shock syndrome | 0.01734 | ELANE |
| Thrombophilia | 0.01734 | ELANE |
| Anti-basement membrane glomerulonephritis | 0.01734 | XCL1 |
| Goodpasture syndrome | 0.01734 | XCL1 |
| Neutropenia | 0.01773 | ELANE |
| Agranulocytosis | 0.01849 | ELANE |
| Pulmonary emphysema | 0.02193 | ELANE |
| Extrinsic asthma | 0.02231 | XCL1 |
| Allergic asthma | 0.02231 | XCL1 |
| IgA glomerulonephritis | 0.02498 | XCL1 |
| Adult respiratory distress syndrome | 0.02574 | ELANE |
| Chronic asthma | 0.02878 | XCL1 |
| Commensal bacterial infectious disease | 0.03106 | ELANE |
| COVID-19 | 0.03143 | ELANE |
| Respiratory failure | 0.03899 | ELANE |
| Acute myocardial infarction | 0.04012 | ELANE |
| Blood platelet disease | 0.04725 | SLC7A11 |
| Middle cerebral artery infarction | 0.04837 | ELANE |
| **Downregulated** |  |  |
| **Other diseases**  Opportunistic mycosis | 0.00501 | STATH/CSF2 |
| Systemic mycosis | 0.00628 | STATH/CSF2 |
| Fungal infectious disease | 0.01405 | STATH/CSF2 |
| Juvenile myelomonocytic leukemia | 0.01637 | CSF2 |
| Pneumocystosis | 0.01637 | CSF2 |
| Adenoma | 0.01768 | HCRTR2/DEFA6/HSD3B2 |
| Myelodysplastic/myeloproliferative neoplasm | 0.01961 | CSF2 |
| Achalasia | 0.01961 | NOS1 |
| Allergic disease | 0.02149 | NOS1/CSF2 |
| Hyperprolactinemia | 0.02607 | HSD3B2 |
| Nephrosis | 0.02761 | NOS1/CCL13 |
| Visceral leishmaniasis | 0.02768 | CSF2 |
| Autosomal recessive congenital ichthyosis | 0.02929 | ABCA12 |
| Pulmonary alveolar proteinosis | 0.02929 | CSF2 |
| Interstitial cystitis | 0.02929 | CSF2 |
| Schistosomiasis | 0.02929 | CSF2 |
| Hepatic encephalopathy | 0.03249 | NOS1 |
| Parasitic protozoa infectious disease | 0.03400 | NOS1/CSF2 |
| Atopic dermatitis | 0.03577 | CSF2/CCL13 |
| Pancreatitis | 0.03649 | NOS1/PPY |
| Allergic contact dermatitis | 0.03904 | CSF2/CCL13 |
| Contact dermatitis | 0.04016 | CSF2/CCL13 |
| Candidiasis | 0.04046 | STATH |
| Pancreas disease | 0.04054 | NOS1/PPY |
| Cell type benign neoplasm | 0.04191 | HCRTR2/DEFA6/HSD3B2 |
| Hypospadias | 0.04204 | HSD3B2 |
| Ichthyosis | 0.04362 | ABCA12 |
| Parasitic infectious disease | 0.04514 | NOS1/CSF2 |
| Retinopathy of prematurity | 0.04520 | NOS1 |
| Lissencephaly | 0.04993 | NOS1 |
| Renovascular hypertension | 0.04993 | NOS1 |
